# Initial clinical experience with a radiation oncology dedicated open 1.0T MR‐simulation

**DOI:** 10.1120/jacmp.v16i2.5201

**Published:** 2015-03-08

**Authors:** Carri K. Glide‐Hurst, Ning Wen, David Hearshen, Joshua Kim, Milan Pantelic, Bo Zhao, Tina Mancell, Kenneth Levin, Benjamin Movsas, Indrin J. Chetty, M. Salim Siddiqui

**Affiliations:** ^1^ Department of Radiation Oncology Henry Ford Health System Detroit Michigan; ^2^ Department of Radiology Henry Ford Health System Detroit Michigan USA

**Keywords:** MR simulation, quality assurance, MRI, distortion characterization

## Abstract

The purpose of this study was to describe our experience with 1.0T MR‐SIM including characterization, quality assurance (QA) program, and features necessary for treatment planning. Staffing, safety, and patient screening procedures were developed. Utilization of an external laser positioning system (ELPS) and MR‐compatible couchtop were illustrated. Spatial and volumetric analyses were conducted between CT‐SIM and MR‐SIM using a stereotactic QA phantom with known landmarks and volumes. Magnetic field inhomogeneity was determined using phase difference analysis. System‐related, in‐plane distortion was evaluated and temporal changes were assessed. 3D distortion was characterized for regions of interest (ROIs) 5–20 cm away from isocenter. American College of Radiology (ACR) recommended tests and impact of ELPS on image quality were analyzed. Combined ultrashort echotime Dixon (UTE/Dixon) sequence was evaluated. Amplitude‐triggered 4D MRI was implemented using a motion phantom (2–10 phases, ~2 cm excursion, 3–5 s periods) and a liver cancer patient. Duty cycle, acquisition time, and excursion were evaluated between maximum intensity projection (MIP) datasets. Less than 2% difference from expected was obtained between CT‐SIM and MR‐SIM volumes, with a mean distance of <0.2 mm between landmarks. Magnetic field inhomogeneity was <2 ppm. 2D distortion was <2 mm over 28.6–33.6 mm of isocenter. Within 5 cm radius of isocenter, mean 3D geometric distortion was 0.59±0.32 mm (maximum=1.65 mm) and increased 10–15 cm from isocenter (mean=1.57±1.06 mm, maximum=6.26 mm). ELPS interference was within the operating frequency of the scanner and was characterized by line patterns and a reduction in signal‐to‐noise ratio (4.6–12.6% for TE=50−150 ms). Image quality checks were within ACR recommendations. UTE/Dixon sequences yielded detectability between bone and air. For 4D MRI, faster breathing periods had higher duty cycles than slow (50.4% (3 s) and 39.4% (5 s), p<0.001) and ~ fourfold acquisition time increase was measured for ten‐phase versus two‐phase. Superior–inferior object extent was underestimated 8% (6 mm) for two‐phase as compared to ten‐phase MIPs, although <2% difference was obtained for ≥4 phases. 4D MRI for a patient demonstrated acceptable image quality in ~7 min. MR‐SIM was integrated into our workflow and QA procedures were developed. Clinical applicability was demonstrated for 4D MRI and UTE imaging to support MR‐SIM for single modality treatment planning.

PACS numbers: 87.56.Fc, 87.61.‐c, 87.57.cp

## I. INTRODUCTION

Historically, magnetic resonance imaging (MRI) has been integrated into radiotherapy treatment planning primarily as an adjunct to computed tomography (CT) to assist in tumor delineation for many treatment sites such as brain, liver, head and neck, and prostate. This conventional CT‐based workflow relies on target and/or organ at risk (OAR) definition on the MRI, and a transfer of contours to CT via image registration for subsequent treatment planning. However, performing MRI to CT image registration introduces additional systematic uncertainties (typically ~2 mm) that can be detrimental to localization of target and organs at risk.^1^, ^2^ Furthermore, having two separate simulations burdens the clinical workload and the use of CT‐SIM exposes the patient to ionizing radiation. Thus, implementing MRI as a stand‐alone simulation modality (i.e., MR‐SIM) for radiation therapy treatment planning is advantageous. To this end, others have implemented radiation oncology dedicated MR scanners, including characterization of low‐field MR, and integration of data into treatment planning for delineation and dose calculation.^3^, ^4^, ^5^ For example, Mah et al.^(4)^ described their implementation of 0.23T MR‐SIM, including external lasers used for virtual isocenter and distortion quantification and correction. Kapanen et al.^(6)^ described their commissioning process for MRI‐only treatment planning at 1.5T, although their work was focused specifically on implementation for prostate cancer. Integrated platforms between the MRI and linear accelerator, often incorporating patient trolley systems to assist with similar setup between imaging and treatment, have also been described.^7^, ^8^, ^9^

Recently, dedicated MR‐SIM platforms have been introduced, adding features to further improve integration into radiation therapy, such as flat tabletops, external laser systems that interface with scanner software, and dedicated radiation therapy imaging protocols. Here, we describe our initial experience with one of the only radiation therapy‐dedicated MR‐SIM platforms clinically available.^(10)^ While other groups^4^, ^6^, ^11^, ^12^ have reported on their implementation of different components of MRI as a simulator at other field strengths, we add to the literature by describing our initial clinical experience with a dedicated 1.0T open dedicated MR‐SIM platform with a vertical magnetic field design. Specifically, we describe our personnel requirements and safety procedure, initial MR‐SIM characterization including distortion quantification, establishment of a quality assurance (QA) program, immobilization devices, and specialized imaging sequences such as 4D MRI and ultrashort echotime (UTE) and Dixon imaging for motion management and bone segmentation, respectively. This work characterizes one of the only commercially available radiotherapy‐dedicated MR‐SIM platforms currently available and, coupled with ongoing work generating synthetic CTs^(13)^ for dose calculation and the characterization of object‐induced distortions, is a first step toward the overarching goal of using MR‐SIM for single modality simulation at our institution.

## II. MATERIALS AND METHODS

### A. Safety and personnel requirements for MR‐SIM

**Figure 1:**
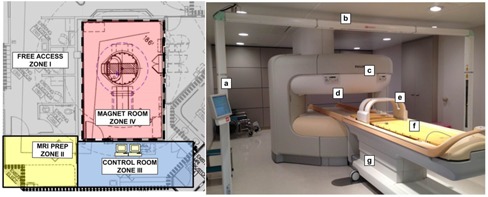
(Left) Schematic of our MR‐SIM suite and corresponding four‐zone regions and their uses as recommended by the American College of Radiology. (Right) 1.0 T Philips Panorama High Field Open (HFO) magnetic resonance system radiation oncology dedicated MR‐SIM and its components: (a) external laser positioning system in‐room console, (b) external laser positioning system (bridge), (c) vertical field magnet, (d) scanner's integrated laser (i.e., light visor), (e) large body coil with solenoid technology, (f) MR‐compatible indexed couchtop, and (g) patient support system trolley.

In the design of the MR‐SIM suite, we followed the American College of Radiology (ACR) “four zone” concept — to restrict the areas affected by the magnetic field (i.e., Zones III (control room) and IV (magnet room)),^(14)^ as shown in Fig. 1(left). Zone I is open to the general public and includes any area outside of the MRI environment. Zone II is our MRI preparation area used for MR screening, taking patient histories, and patient dressing. Zone II is the region in between the uncontrolled Zone I and strictly controlled Zones III and IV. Patients do not move from Zone II to Zone III unless under the supervision of Level 2 MR personnel or those who have been trained to ensure MR safety guidelines are strictly adhered to for patient, personnel, and equipment safety.^(14)^ Our current Level 2 MR personnel consist of MR technologists, diagnostic medical physicists, and MR radiologists. By contrast, Level 1 personnel include those who have passed minimal safety educational requirements to ensure their safety as they work in Zone III, such as our radiation oncologists, therapy medical physicists, radiation oncology nursing staff, radiation therapists, and researchers. According to the ACR, while Level 1 personnel are allowed unaccompanied access to Zones III and IV, they cannot be responsible for non‐MR personnel in Zone IV. Our MR‐SIM team is led by: 1) a board‐certified MR physicist who assisted with machine acceptance, quality assurance (QA), parameter optimization for clinical protocols, and MR safety training, and 2) a board‐certified MR radiologist who provides qualitative feedback on image quality and acquisition sequences.

### B. Safety screening and patient workflow

Efforts are necessary to develop and administer patient and personnel screening documentation and policies. In our institution, we adhere to existing policies developed by our radiology department that are similar to what has been outlined in the literature.^(14)^ Screening includes evaluating MR eligibility and the presence of contraindications, such as implanted metal objects/implants or nonremovable body piercings, pregnancy, claustrophobia, and inability to lie still for >30 min. The ACR recommends a minimum of two separate MR safety screening sessions conducted by two people, one of which is Level II MR personnel.^(14)^ At our institution, patients are screened on three occasions: 1) initially, before the MR‐SIM appointment has been scheduled to ensure eligibility, 2) the day of the MR‐SIM, often in the radiation oncology department, and 3) in Zone II (i.e., before entering Zone III). We typically have our radiation oncology nursing staff conduct the initial screening, our radiation therapist conduct the second screening the day of the procedure, and the MR technician conduct the final screening before the patient is scanned to ensure the most conservative approach to patient safety.

Due to limitations in the current version of the MR‐SIM software (HFO RT Oncology Configuration, v3.5.2), we are unable to mark the final isocenter (setup point) using MR images. This functionality, including the transfer of isocenter coordinates to the scanner and laser system for subsequent patient marking, is expected in a future upgrade. Nevertheless, it is possible to use a reference point marking approach, as described in TG‐66,^(15)^ where MR‐compatible skin markers and reference marks could be used. Given the current limitations in the software, our current workflow includes performing the CT‐SIM and MR‐SIM on the same day, with the CT‐SIM preceding the MR‐SIM appointment. We set isocenter and conduct patient marking using CT‐SIM, and then transfer the isocenter coordinates to the MR‐SIM LAP laser software (described in Materials & Methods section C.2). The MR‐SIM lasers will then move to the appropriate sagittal and lateral locations from the marked CT‐SIM isocenter, and the immobilized patient can be positioned accordingly. By importing the CT‐SIM isocenter in this manner, patients can then be translated by a known physical offset to the magnet's isocenter, thereby introducing the least amount of distortion in the anatomy of interest. With added functionality, future work will involve exploring MR‐SIM for patient marking.

An MR‐SIM Time Out checklist is also administered before the beginning of scanning to ensure patient safety. The Time Out checklist is based on the “Universal Protocol” recommended by the Joint Commission to prevent surgical errors^(16)^ and has been adapted for our imaging and radiation therapy process to ensure proper patient identity, treatment site, MRI safety screenings, patient positioning, presence of two hearing protection devices (i.e., ear plugs and headphones), and absence of skin folds and patient loops (i.e., skin‐to‐skin contact in hands touching) and that the lasers are turned off (see Materials & Methods section H.2.1) before scanning procedures are initiated.

### C. MR simulator and auxiliary components

#### C.1 MR simulator and software

The 1.0 T Philips Panorama High Field Open (HFO) Magnetic Resonance System (Philips Medical Systems, Cleveland, OH) (MR‐SIM) was installed in January, 2013 and acceptance testing was performed in March, 2013. The system consists of a vertical magnetic field design with 160 cm wide aperture and a 45 cm field of view (Fig. 1(right)), which enables lateral table translation of up to 28 cm to center the anatomy of interest at the magnet isocenter. Because of the vertical field design, dedicated coils with integrated solenoid technology (Fig. 1(e) for the large body coil) are used with the receiver elements perpendicular to the body's long axis. Another benefit of the rigid coils is that many MR‐compatible immobilization devices can be accommodated while maintaining the patient's external contour for treatment planning purposes.

The MR‐SIM has integrated software that includes QA imaging sequences (distortion correction, laser QA), functionality to swap the laser system (described in detail later), and initial radiation therapy Exam Cards (i.e., preprogrammed image acquisition settings) for brain, pelvis, head and neck, and gynecological cases.

#### C.2 Laser systems

The MR‐SIM consists of two laser systems: an integrated laser (Fig. 1(d), also called the “light visor”) and MR‐compatible external laser positioning system (ELPS) (Fig. 1(b)). The integrated light visor laser is a Class II, 635 nm laser at the end of the bore used to select the plane that will be positioned in the center of the magnet and to assist with patient positioning. The ELPS DORADOnova MR3T (LAP of America Laser Applications, Boynton Beach, FL) consists of six Class II sagittal, transverse, and coronal external lasers used for patient positioning and translational/rotational alignment. The added external lasers are to enable correlation between external skin marks and MR images. The ELPS is similar to the CT‐SIM laser system where the patient is localized using a virtual isocenter outside of the magnet bore,^(15)^ although the MR‐SIM software does not currently allow for interactive isocenter marking using patient images. The MR console is configured to use the ELPS system for patient positioning, although this communication must be manually enabled on a daily basis. If the patient has undergone a CT‐SIM with isocenter marking, the isocenter coordinate information can be imported from CT‐SIM into the ELPS software and loaded. The lasers will then move to the appropriate sagittal and lateral locations from the marked isocenter, and the patient can be positioned accordingly. It is important to note that, while the patient support system trolley can move vertically, the couch does not adjust in the vertical direction once it is inside the bore. Finally, while patients can be localized using the ELPS, the table will be laterally translated to move the anatomy of interest to the isocenter, which is particularly important for lateral lesions such as breast or extremities. During patient scanning, the ELPS must be disabled, as it degrades image quality, as evaluated in Materials & Methods section H.2.1.

#### C.3 MR‐compatible couchtops

The Indexed Patient Positioning System (IPPS Overlay, CIVCO Medical Solutions, Kalona, IA) is a flat couch overlay device with indexing capability with two different sizes: 40 cm width for upper body imaging, and 50 cm width for pelvic/lower body imaging. An MR‐compatible Lok‐Bar (CIVCO Medical Solutions, Kalona, IA) consists of three pins to enable consistent positioning of immobilization devices between MR‐SIM and CT‐SIM/radiation therapy. As shown in Fig. 1(f), the couchtop sits on the patient support system on top of risers that allow the couch to be 18.5 mm above the MR‐SIM patient support system and coils, thereby preventing the coils from touching the patient or immobilization devices. The IPPS weight limit is 250 kg (550 lbs) and requires two personnel to affix it to the integrated patient support system.

### D. Volumetric analysis

**Figure 2:**
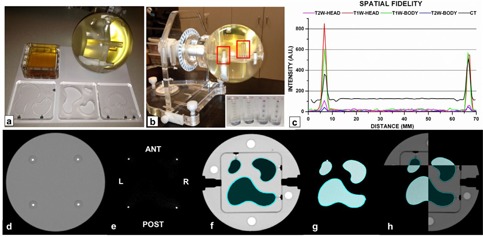
Lucy 3D QA phantom used for volumetric, spatial, and registration comparisons between CT‐SIM and MR‐SIM. (a) Clockwise from the top left: phantom signal generator, Lucy phantom, MR and CT phantom inserts; (b) setup of the Lucy phantom using the manufacturer‐provided leveling base (red boxes=placement of CT marker cylinders used for spatial fidelity testing; inset: MRI marker cylinders consisting of 2 mm oil beads (two left) and CT marker cylinders with 2 mm aluminum beads used for spatial testing (two right)); (c) line profile analysis of the T1‐weighted (T1W) and T2‐weighted (T2W) datasets acquired using the head and body coils and the CT‐SIM where the peaks indicate marker locations; (d) CT‐SIM of the CT marker cylinders; (e) axial T1‐weighted MR‐SIM scan of the marker cylinders; (f) axial CT‐SIM scan of the volumetric inserts; (g) axial T1‐weighted MR‐SIM scan of the volumetric inserts; and (h) image fusion of the CT‐SIM andT1‐weighted MR‐SIM datasets. ANT=anterior, POST=posterior, L=left, R=right.

To compare volumes between CT‐SIM and MR‐SIM, the LUCY 3D Plus QA phantom (Standard Imaging, Middleton, WI) (Fig. 2(a)) was employed. The Lucy phantom is a high‐precision, MR‐compatible modular device that is specifically designed to meet the needs required for stereotactic QA.^(17)^ For both MR‐SIM and CT‐SIM acquisitions, the Lucy phantom was affixed to its precision leveling base (Fig. 2(b)). Briefly, for MRI scanning, an MRI signal generator (i.e., cavity filled with manganese chloride solution) was first fitted into the Lucy phantom to produce appropriate levels of MR signal strength, as shown in Fig. 2(a). An MRI volumetric insert containing three irregular shapes filled with mineral oil and known volumes was then imaged — these volumes were considered the “true volumes” based on the manufacturer‐stated volume. MR‐SIM scans were acquired with both a four element, phased‐array head coil and a two element, phased‐array medium body/spine coil. Two frequently used clinical sequences were used: a 3D T1‐weighted fast‐field echo acquisition sequence ($\text{TE}/\text{TR}/\alpha =6.9/25\;\text{ms}/30^{{}^{\circ}}$ and pixel bandwidth=112 Hz/pixel, and a 3D T2 turbo‐spin echo sequence ($TE/TR/\alpha =90/3683\;ms/90^{{}^{\circ}}$ and pixel bandwidth=150 Hz/pixel). Both sequences used the following parameters: $\text{FOV}=223\times 231\times 40\;{\text{mm}}^{3}$, acquisition $\text{matrix}=332\times 323\;{\text{mm}}^{2}$, and $\text{voxel size}\approx 0.69\times 0.69\times 1\;{\text{mm}}^{3}$. For CT‐SIM acquisition, a clinical stereotactic radiosurgery brain acquisition protocol was used with 1 mm axial slice thickness, 512×512 image dimension, 120 kVp, 284 mAs, and 0.68×0.68 mm pixel spacing.

All MR‐SIM and CT‐SIM phantom data were transferred to the Eclipse Treatment Planning System (Eclipse TPS, v11.0, Varian Medical Systems, Palo Alto, CA) for subsequent analysis. To standardize object contouring, small, medium, and large irregular regions of interest (ROIs) were segmented using a set window/level for each modality and automated thresholding in Eclipse, as illustrated in Figs. 2(f) and (g), for the CT‐SIM and MR‐SIM, respectively. Volume percent differences were calculated using known manufacturer‐stated true volumes.

### E. Spatial fidelity and image registration

Four marker cylinders, each containing five 2 mm oil beads (MRI) or five 2 mm aluminum beads (CT) (Fig. 2(b)), were fit into precision‐machined cavities into one hemisphere of the Lucy 3D QA Phantom. One end of the cylinders has red etching for orientation purposes, and the oil beads and aluminum beads are spaced 5 mm center to center. The marker cylinders are oriented in a known, rigid geometry forming a rectangle of 60 mm center to center. Spatial fidelity was evaluated by generating line profiles across (right to left) the marker cylinders in the axial plane for the CT‐SIM, T1‐weighted, T2‐weighted, and head/body coil datasets, as shown in Figs. 2(c) to (e). The peaks of the implanted cylinders were determined by finding the local maximum intensity for each marker via a bounding box and search function (OriginLab, version 6.1, OriginLab Corporation, Northampton, MA). Peak‐picking was visually verified and modified to refect the center of the peak, if necessary. Absolute displacement and percent differences from the ground truth were calculated for both CT‐SIM and MR‐SIM datasets for the four locations (anterior, posterior, left, and right, as shown in Fig. 2(c)). MR‐SIM to CT‐SIM image registration was performed in the Eclipse Registration Workspace using automatic, intensity‐based image registration (rotation/translation) that employs a linear optimization method with mutual information calculation.^(18)^ The registration results between the CT and MRI marker cylinders were visually inspected for agreement.

### F. Initial magnetic field homogeneity characterization

At time of acceptance, the initial magnetic field homogeneity was characterized using an implemented phase difference analysis technique recommended by the ACR.^(19)^ Briefly, a 31 cm diameter uniform spherical phantom was imaged with the quadrature body coil. Two‐dimensional (2D) gradient echo images were acquired in axial, sagittal, and coronal planes using a large FOV covering the phantom with the following parameters: $\text{TE}1/\text{TE}2/\text{TR}/\alpha =10/12/500\;\text{ms}/20^{{}^{\circ}}$, pixel bandwidth=1420 Hz/pixel, $\text{acquisition matrix}=176\times 88\;{\text{mm}}^{2}$, $\text{FOV}=350\;{\text{mm}}^{2},\text{voxel size}\approx 0.5\times 0.5\times 5{\text{mm}}^{3}$. Phase difference maps were generated between the two TE datasets using in‐house software to determine the pixel‐by‐pixel measurement of field homogeneity for each axis using the phase images. Images were normalized between 0 and 2π and transition zones (i.e., areas where the original phase variation exceeded 2π) were removed using a standard phase unwrapping algorithm.^(20)^ A phase difference map was generated by subtracting the phase image at TE=10 ms from that at TE=12 ms to yield phase difference values directly proportional to the magnetic field at each pixel.^(21)^

### G. Geometric fidelity

Geometric accuracy can be classified into two major components: system‐related distortions (magnetic field distortions and gradient nonlinearity) and patient/object‐induced distortions (e.g., chemical shifts and susceptibility). Geometric distortion due to magnetic field inhomogeneity and gradient field nonlinearity has been well‐documented in the literature,^22^, ^23^, ^24^, ^25^, ^26^, ^27^, ^28^ although not specifically for the 1.0T HFO. Two‐dimensional in‐plane distortion is evaluated for routine daily QA using a vendor‐provided geometric planar distortion phantom scanned in three planes (axial, sagittal, and coronal) using the integrated quadrature body coil. A vendor‐supplied imaging protocol generates images over ~35 cm×40 cm field of view (FOV) and 2D distortion is quantified using vendor‐provided software. The software compares the acquired data to an ideal grid of phantom markers, and deviations between the expected and measured values based on center of mass analysis. Two‐dimensional contours are then interpolated to the acquired images based on the calculated deviations, rendering a distortion plot with isocontours ranging from 2 to 6 mm for each plane. A rectangular ROI is automatically derived indicating the area where >75% of the distortion is <2 mm. The MR technician visually verifies that the location of the 2 mm isocontour does not extend substantially into the rectangular region, and records the dimensions of the rectangular ROI. In this manner, 2D distortion can be assessed on a routine basis, before the MR‐SIM is used for daily operation, and temporal changes can be ascertained.

**Figure 3:**
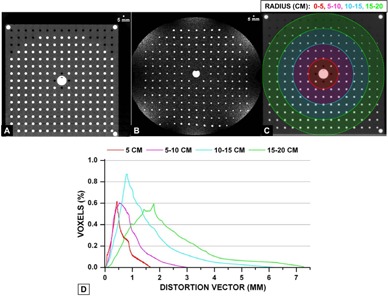
(a) Axial CT scan of the phantom demonstrating the capsule array, (b) 1.0T axial scan at similar slice location, (c) region of interests from isocenter used for distortion analysis between MR‐SIM and CT‐SIM, and (d) distortion vector analysis between MR‐SIM and CT‐SIM at the different radii.

Three‐dimensional (3D) distortion characterization was performed during MR‐SIM acceptance using a prototype phantom provided by the vendor. Because of the large phantom size (40 cm×40 cm×40 cm), the integrated quadrature body coil was used for scanning. The phantom consisted of docusate sodium capsules (~12 mm length, 6 mm diameter, Fig. 3(a)) with 2.5 cm centroid‐to‐centroid, in‐plane spacing and 2.7 cm z‐axis centroid‐to‐centroid spacing, yielding ~2500 control points over the phantom volume. The phantom was first scanned with CT‐SIM (Philips Big Bore, Philips Medical Systems) with $600\;{\text{mm}}^{2}$ FOV, 2 mm axial slice thickness, 120 kVp, 284 mAs, $\text{voxel size}\approx 1.19\times 1.19\;{\text{mm}}^{2}$), as shown in Fig. 3(a)). Three‐dimensional T1 fast‐field echo acquisitions were acquired with the 1.0 T MR‐SIM ($\text{TE}/\text{TR}/\alpha =3.83/9\;\text{ms}/10^{{}^{\circ}}$, pixel bandwidth=191 Hz/pixel, $\text{voxel size}\approx 0.938\times 0.938\times 1{\text{mm}}^{3}$, acquired using a FOV of $450\times 450\times 400{\text{mm}}^{3}$), as shown in Fig. 3(b).

To derive a 3D distortion map, deformable image registration (DIR) was conducted using Velocity Advanced Imaging (VelocityAI, v3.0.0, Velocity Medical Solutions, Atlanta, GA). We have previously benchmarked VelocityAI DIR using 11 computational models developed via finite element methods (FEM) from patient lung CT scans with simulated motion ranging from 1.8 cm to 3 cm.^(29)^ FEM‐generated displacement vector fields (DVFs) served as the gold standard and mean errors were 1.0~3.0 mm for the computational phantoms, with regions of large displacements yielding larger registration errors. Extrapolating these results to much smaller distortion magnitudes expected in MR‐SIM, <1 mm errors can be expected. Because the phantom was in slightly different positions between MR‐SIM and CT‐SIM, manual matching and automated rigid registration (1 pass) were performed. Then, deformable (1 pass) registration was implemented between the MR‐SIM and CT‐SIM data. With a perfect DIR, the deformed image should be exactly the same as the fixed image. Visual verification of the congruence of the MR‐SIM and CT‐SIM images was assessed through an overlay and checked via the split view, blending view, and spyglass tools available in VelocityAI, which is consistent with our clinical practices.^(30)^ Image congruence was found to improve when the MR‐SIM data was used as the primary dataset. When CT‐SIM was used as the primary dataset, the larger FOV caused erroneous expansion of the MR‐SIM volume at the image boundaries. In addition, the MR‐SIM data showed some signal loss at the boundary (Fig. 3(b)), which may also have affected the DIR performance.

DVFs between the MR‐SIM and CT‐SIM data were used to describe the vector distortion magnitude. Distortion histograms (i.e., percent volume versus vector distortion magnitude) for several ROIs around isocenter were derived (5 cm radius sphere, 5–10 cm radius annulus, 10–15 cm radius annulus, and 15–20 cm radius annulus, as shown in Fig. 3(c) and (d)).

### H. Daily quality assurance procedures

Daily quality assurance procedures are all conducted by the MR technician on days that the MR‐SIM is in clinical or research use.

#### H.1 Image quality assessment

For ongoing daily quality assurance, we use a manufacturer‐provided Plexiglas phantom (200 mm diameter, 110 mm length) containing fluid with known relaxation time accuracy of ±5%. Vendor‐supplied PIQT software is used that includes modules for all of the weekly ACR performance tests, including automated measures of table positioning accuracy, central (i.e., resonant) frequency, and transmitted gain. Image quality metrics are assessed using National Electrical Manufacturers Association (NEMA) standards that were developed to enable comparisons across other MR systems.^31^, ^32^ Tests included spatial resolution, low‐contrast detectability, food‐field uniformity, and artifact detection (e.g., ghosting or lines). The PIQT also includes annual ACR QA recommendations, such as slice thickness accuracy (full width half maximum (FWHM) of a 1 mm thick standard crossed‐ramp phantom insert (~11° angle)) and integral uniformity. Results are monitored daily by the MR technician (see Appendix A) and on a monthly basis by the physicist performing monthly QA.

#### H.2 Laser quality assurance

##### H.2.1 Isocenter offset and alignment

An offset between the external bridge lasers and the magnet isocenter is determined at time of commissioning and verified before daily use, in a manner analogous to CT‐SIM. The LAP Aquarius MR phantom (Laser Alignment (LA phantom); LAP of America Laser Applications) and three‐point leveling platform for routine laser QA are used for daily laser QA. The phantom has copper sulfate signal generators and a 2 mm thick, 15 cm long by 15 cm wide oil‐filled internal crosshair that is imaged in each plane. Etched scribes on the external phantom surface coincide with the internal crosshair. The leveling platform affixes to the IPPS table and phantom grooves are aligned to the sagittal, transverse, and coronal external LAP lasers for translational and rotational alignment. The body coil is connected over the top of the phantom, and the phantom is automatically translated to the magnet isocenter inside the bore using the established offset. Twenty‐four images (8 in each orientation) are acquired using 2.0 mm slice thickness, 0 mm gap, and 200 mm field of view based on a vendor‐supplied protocol. The offsets are then characterized by assessing the deviations of the internal crosshair from the origin in the anterior–posterior, right–left, and foot–head directions. A similar procedure is conducted to test the known longitudinal offset of the light visor laser (i.e., scanner's integrated laser).

Offsets of the internal crosshair from the implanted markers are recorded in all directions and are expected to be <2 mm.

##### H.2.2 Laser impact on image quality

Because the ELPS is positioned inside the MR room, when powered on, the system produces radiofrequency interference that generates MR image artifacts. To characterize the impact on image quality, two separate tests were conducted: a spurious noise test and the PIQT test with and without the ELPS powered on. For the spurious noise test, a service protocol was run that isolates and detects other frequencies outside of the scanner. Images were acquired using the integrated quadrature body coil. For the PIQT test, image‐quality parameters were automatically evaluated using built‐in clinical software, including food field uniformity, SNR, spatial linearity, slice profile, and spatial resolution. It should be noted that evaluating SNR may not be appropriate for images containing known artifacts;^(33)^ however, it is instructive to verify that the established QA procedures will identify and prevent leaving the ELPS powered on during patient scanning. In addition, we have established a workflow in our Time Out procedure to ensure the ELPS has been powered off prior to patient scanning.

### I. Monthly quality assurance procedures

#### 1.1 Image quality

Our monthly QA procedures include scanning of a large ACR accreditation phantom to determine the minimum levels of performance for well‐functioning MRI systems.^(34)^ The phantom contains seven image quality modules including: geometric accuracy, high‐contrast spatial resolution, slice thickness and position accuracy, slice position accuracy, image intensity uniformity, percent signal ghosting, and low‐contrast object detectability. Results are compared with those acquired at time of acceptance testing. We also monitor the central frequency over time.

#### 1.2 Laser motion and alignment

We follow the recommendations provided by AAPM TG‐66 for laser motion and alignment monthly QA.^(15)^ The ELPS laser movement should be accurate, linear, and reproducible. The LA phantom is first set up and leveled. The phantom has machined grooves that are aligned to the ELPS transverse, sagittal, and coronal lasers. To verify that the light visor laser and ELPS lasers are parallel and orthogonal to the scan plane, the couchtop is moved longitudinally and laterally while the lasers are observed to ensure deviations from the phantom grooves are less than ±2 mm.

To verify the ELPS individual side laser movement, a calibrated ruler is taped to the side of the LA phantom. The vertical (Z) position of the side laser on the ruler is recorded. The laser is then moved by 200 mm using the in‐room control monitor. The position is recorded and compared to the expected with a tolerance of 1 mm. The lasers are then sent back to the zero position, and the test is repeated for the other side laser. For the sagittal ELPS laser, the calibrated ruler is placed on the couchtop perpendicular to the sagittal laser. The sagittal laser is moved to +100 mm and −100 mm, and the position of the laser on the ruler is recorded. The measured values should be within ±1 mm of expected values.

### J. Initial patient experience

#### J.1 Immobilization devices

One of the first tasks for patient scanning included identifying immobilization devices that are considered “MR Compatible” according to guidelines set forth by the Food and Drug Administration.^(35)^ Our MR‐SIM patient experience has primarily included prostate, spine, and brain cases. Table 1 demonstrates the coils, immobilization devices, and typical MR‐SIM sequences acquired. For lumbar spine, the exam time was quite long for 3D scanning and patient motion could sometimes compromise image quality. Thus, we have moved forward with 2D protocols for this treatment site to decrease scan time. In addition, we enable sensitivity encoding (SENSE factor of typically 1.3~2.0), whenever possible, to shorten exam times and reduce motion artifacts. Typical exam times are 4−7 min per scan type, although they largely depend on the field of view selected. For thoracic spine, we are still refining our imaging techniques to acquire high‐quality images in the presence of respiratory motion.

**Table 1:** Site‐specific coils, immobilization, and imaging sequences optimized for MR‐SIM implementation

*Patient Case*	*Imaging Coil*	*Immobilization Devices*	*Typical Sequences Acquired*
Pelvis	• Body coil:	• Square sponge under head	Whole pelvis
• Prostate	Medium‐Extra Large	• Angled sponge under knees/legs	• 3D T1‐weighted Fast Field Echo
• Gynecological	• Integrated quadrature coil		• 3D T2‐weighted Turbo Spin Echo Cone‐down (higher resolution)
		• Banded feet	
		• Hands holding ring	• T2‐weighted Turbo Spin Echo
Brain	• Head coil	• Blue CIVCO Type‐S	• 3D T1‐weighted
		Overlay board	• 3D T2‐weighted Turbo Spin Echo
		• CIVCO 5‐point mask	• Axial T2‐weighted Axial FLAIR
		• Headrest “B” (clear /Silverman)	
		• Angled sponge under knees/legs	
		• Banded feet	
		• Hands holding ring	
Cervical Spine	• Head coil	• Blue BodyFix bag (not pump)	• 3D T1‐weighted
	• Body coil:		• 3D T2‐weighted Turbo Spin Echo
	Medium‐Extra Large	• Half circle leg sponge	• VISTA CLEAR
	• Integrated quadrature coil	• Arms at sides	• Balanced Fast Field Echo for head and neck region
		• Feet neutral	
Lumbar Spine	• Body coil:	• Blue BodyFix bag (not pump)	• 2D T1‐weighted
	Medium‐Extra Large		• 2D T2‐weighted Turbo Spin Echo
	• Integrated quadrature coil	• Half circle leg sponge	
		• Arms at sides	
		• Feet neutral	

All images obtained with the first ten patients scanned with the MR‐SIM were reviewed by a board‐certified radiologist (M.P.) for qualitative image assessment. Feedback was provided from the radiologist and sequences were iterated and added as recommended.

#### J.2 Ultrashort echotime (UTE) and Dixon techniques

Historically, it has been difficult to discriminate between cortical bone and air in MRI images due to the extremely short T2 and T2* relaxation times and fast‐decaying signals. One solution includes employing ultrashort echotime (UTE) sequences to assist in cortical bone segmentation.^36^, ^37^, ^38^ Using UTE sequences to generate synthetic CTs for treatment planning purposes^39^, ^40^ or for combined PET/MRI units^41^, ^42^ has shown great promise. For tissue classification, such as distinguishing lipids (i.e., fat) from water, Dixon imaging has also proven useful.^39^, ^41^, ^43^, ^44^, ^45^ We have implemented a combined ultrashort echotime (UTE) and Dixon (i.e. UTE/Dixon) image sequence on our 1.0T MR‐SIM under an IRB‐approved prospective imaging protocol. The novel two‐point Dixon has been shown to improve fat suppression, spatial resolution, and image quality in the abdomen compared to standard techniques.^45^, ^46^ Advantages of combining Dixon and UTE into one image sequence include shortened exam time and reduced image registration uncertainties between datasets. The UTE/Dixon sequence includes a single triple‐echo scan (one free‐induction decay and two echoes for Dixon) acquired using a 3D center‐out radial acquisition mode to enable the shortest TE time possible.

For initial implementation, 3D volumetric UTE/Dixon images of a porcine shank were acquired with a four element phased‐array head coil with the following parameters: TR=8.14 ms, TE=0.144 (UTE), 2.44, and 4.74 ms, flip angle=25°, and $\text{isotropic voxel size}\approx 1.6\times 1.6\times 1.6{\text{mm}}^{3}$, and $\text{FOV}=230\times 230\times 230\;{\text{mm}}^{3}$. The images are used to calculate water and fat components, with an overall scan time of ~5 min for the UTE/Dixon sequence. For comparison purposes, T1‐weighted turbo‐field echo (TR/TE=7.46/3.69 ms, $\text{voxel size}\approx 0.94\times 0.94\times 2.2{\text{mm}}^{3}$) and T2‐weighted (TR/TE=3549/80 ms, flip angle=90°, $\text{voxel size}\approx 0.68\times 0.68\times 3{\text{mm}}^{3}$) were acquired. Using a similar experimental setup, a CT scan was also conducted using slice thickness of 2 mm and in‐plane pixel size of $0.68\;\text{mm}\times 0.68\;{\text{mm}}^{3}\;(512\times 512\;\text{matrix})$. All images were interpolated to match the UTE/Dixon image dimensions for comparison purposes. To further reduce the signal from longer T2 components, a “bone enhanced” image was generated using the Image Algebra postprocessing package available on our scanner. This image was generated by scaling the UTE image by a factor of 1.3 and subtracting the in‐phase image generated by Dixon to further highlight the bone region in the porcine shank. The scaling factor magnitude was determined ad hoc based on the optimal suppression of brain tissue while enhancing the bone. The bone‐enhanced image exhibited a high intensity “halo” of signal around the shank that was removed via image postprocessing (i.e., applying an image mask derived from the outer surface of the T1 dataset and setting the background to white to provide optimal contrast).

UTE/Dixon scans were conducted for a radiosurgery patient status postresection of a left frontal solitary metastasis. The four element phased‐array head coil and immobilization devices listed in Table 1 were used. Three‐dimensional volumetric UTE/Dixon images were acquired using the same parameters as the porcine experiment. For comparison purposes, a post‐Gadolinium enhanced T1‐weighted turbo‐field echo (TR/TE=21/6.9 ms, $\text{voxel size}\approx 0.97\times 0.97\times 2.0{\text{mm}}^{3}$ with a FOV=190 mm (right–left), 233 mm (ant–post), and 164 mm (foot–head) was also acquired. A CT scan was also conducted using slice thickness of 2 mm and in‐plane pixel size of $0.68\;\text{mm}\times 0.68\;{\text{mm}}^{2}\;(512\times 512\;\text{reconstruction matrix})$, and all images were resampled to match the UTE/Dixon image dimensions. A bone‐enhanced image was also generated for the patient case in a manner similar to the porcine shank.

#### J.3 Four‐dimensional MRI (4D MRI)

##### J.3.1 4D MRI Algorithm

Efforts are currently underway to evaluate a respiratory‐triggered, single‐shot T2‐weighted TSE 4D MRI acquisition technique under a prospective IRB‐approved protocol. The algorithm is a multislice 2D dynamic MRI acquisition that was first introduced by Hu et al.^(47)^ Image acquisition of different respiratory states for each MR slice is performed using amplitude‐based triggered acquisition 4D MRI (described in detail in Reference 35). To ensure better image contrast (e.g., by allowing for longer TR times), data sampling is separated into multiple respiratory cycles to allow the magnetization to completely relax. During the 4D MRI acquisition, an external waveform is derived from a clinically available air‐filled cushion and pressure sensor tracking device. The waveform is initially tagged at the start of the magnet's preparation state, and once the preparation state has concluded, a calibration period over the first 2 breathing cycles is analyzed to determine the trigger levels for the different respiratory phases with respect to the maximum and minimum respiratory signal levels obtained. Hu's approach has been further refined to improve clinical efficiency by integrating proprietary measures to reduce acquisition time and stabilize triggering due to breathing anomalies.

##### J.3.2 4D MRI phantom experiment and initial patient result

**Figure 4:**
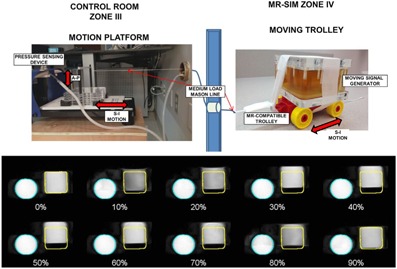
(Top) Experimental setup for 4D MRI phantom study, including the motion platform and components located in the control room that translate the MR‐compatible trolley located in the MR‐SIM bore. (Bottom) Coronal view for a ten‐phase, amplitude‐based triggered 4D MRI for a signal generator phantom translating ~2 cm in the superior–inferior direction (square object, sinusoidal breathing pattern, 3 s period). The percentages describe the breathing phases, with 0% representing end‐inhale and 50% representing end‐exhale. The round static signal generator is also shown in the field of view.

Phantom experiments were carried out using a programmable respiratory motion platform (ExacTrac Gating Phantom, Version 1.0, BrainLAB AG, Feldkirchen, Germany) with string attached that pulled a cube‐shaped signal generator (and placed on a Lego trolley (LEGO Systems A/S, Billund, Denmark) located inside the magnet room (experimental setup shown in Fig. 4). The platform translated the signal generator in the superior–inferior (S‐I) direction. A chest wall component moving simultaneously in the anterior–posterior direction was used to generate a respiratory waveform via a clinically available air‐filled cushion and pressure sensor tracking device (Fig. 4, left). For a stationary reference, a bottle of signal generator (1000 ml bottle of demineralized water/copper sulfate) was scanned in the same FOV. Programmed superior–inferior object excursion was ~3 cm using a sinusoidal waveform with both 3 and 5 s breathing cycles. It should be noted that the actual object excursion was reduced to ~1.5–2 cm due to the addition of the string and trolley system, which is similar to what has been reported in the literature.^(48)^ Two to ten phase T2‐weighted TSE 4D MRI images were acquired with $\text{TE}/\text{TR}/\alpha =50.25/2000\;\text{ms}/90^{{}^{\circ}}$, pixel bandwidth=259 Hz/pixel, voxel size≈0.98×0.98×5 mm, and $\text{FOV}=250\times 201\times 125\;{\text{mm}}^{3}$. All MRI datasets were exported from the scanner in DICOM format. Maximum intensity projections (MIP) were derived using MATLAB (MathWorks, Natick, MA) by calculating the maximum intensity values of all voxels throughout all 4D MRI phases. Duty cycle (nominal programmed acquisition time divided by overall scan time) and excursion were evaluated between phase acquisitions.

A liver cancer patient consented to a prospective IRB‐approved protocol to optimize acquisition parameters for abdominal 4D MRI (6 phases, $\text{TE}/\text{TR}/\alpha =75/6100\;\text{ms}/90^{{}^{\circ}}$, $\text{voxel}\approx 1\times 1\times 7{\text{mm}}^{3}$). Duty cycle, scan time, and image quality were evaluated between phase acquisitions. For image quality comparison purposes, coronal cine‐MRI (~1 frame/s, ~50 s) was also acquired. MIP renderings were generated in a manner similar to the phantom experiment.

## III. RESULTS

### A. Volumetric analysis

CT‐SIM volumes were <2% different from ground truth (maximum difference=−1.8% (0.03 cc) for the smallest insert). T1‐and T2‐weighted images acquired using the head coil, where the signal to noise ratio was appropriate for the size of the object being imaged, were within −1.3% and 2.1% of ground truth, respectively, for the larger inserts. Delineations of the small inserts were slightly larger than expected (1.2−2.4%, 0.02−0.04 cc). In the medium body/spine coil, volumes were consistently smaller than ground truth (−1.7% to −2.7%) for the medium and large inserts. However, for the small insert, volumes were equal or slightly larger for the T2‐ and T1‐weighted images, respectively. Overall, clinically acceptable results were obtained for both CT‐SIM and MR‐SIM (<2% difference from manufacturer‐stated true volumes). The equivalent diameters were <1 mm different for all CT‐SIM and MR‐SIM volumes.

### B. Spatial fidelity and image registration

Figure 2(c) demonstrates the intensity profiles across the CT‐SIM and MR‐SIM cylindrical markers for the different acquisitions and coils. For CT‐SIM, the mean distance between markers was 59.83±0.02 mm (<0.3% difference from expected, 60 mm). For the T1‐weighted and T2‐weighted datasets, the mean distance between markers was 60.05±0.19 mm and 59.99±0.16, respectively, across both coils (<0.4% different from expected). When the head coil and medium body coil were considered separately, the mean distances were 60.07±0.19 and 59.97±0.17 mm, respectively. The image fusion shown in Fig. 2(h)(right) demonstrates the spatial concordance between the MR‐SIM and CT‐SIM images after rigid registration.

### C. Initial magnetic field homogeneity characterization

**Figure 5:**
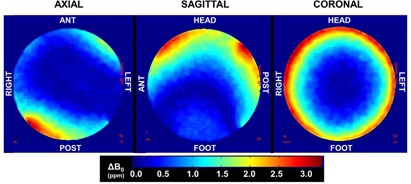
Change in magnetic field inhomogeneity map characterized at time of acceptance. The volume root mean square was calculated to be 0.9 ppm over a 31 cm diameter spherical phantom volume, with an image reconstruction diameter of 35 cm.

Figure 5 demonstrates the magnetic field inhomogeneity maps of the MR‐SIM. The volume root mean square was calculated to be 0.9 ppm over 31 cm diameter spherical volume, which was within ACR specifications. Due to the vertical magnet design, the coronal datasets show the most symmetrical distribution (i.e., left–right, anterior–posterior) because of the equidistance from the magnetic field.

### D. Geometric distortion

**Figure 6:**
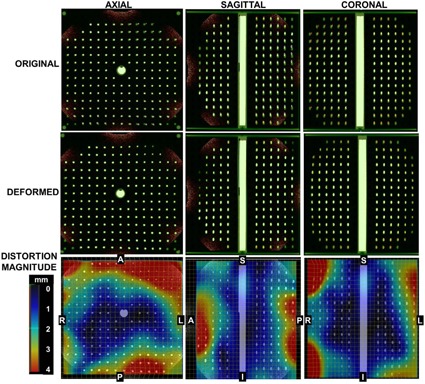
(Top row) Blended overlay of the original CT‐SIM (green) and MR‐SIM data (orange) prior to deformable image registration. Mismatches in marker location can be observed between datasets, particularly near the edges of the field of view. (Middle row) Overlay of CT‐SIM and MR‐SIM data after deformable image registration, showing the resolution of image distortions. (Bottom row) Vector distortion magnitude between the CT‐SIM and MR‐SIM datasets demonstrate the distortions occurring near the edges of the field of view. For reference, centroid‐to‐centroid marker spacing is 2.5 cm in‐plane and 2.7 cm along the z‐axis, and the registration field of view was $\sim 40\times 34\times 40\;{\text{cm}}^{3}$ in the right–left, anterior–posterior, and superior–inferior, respectively.

Two‐dimensional distortion characterization was tracked temporally over the first eight months of operation via a 2D distortion phantom and assessment of rectangular ROIs to indicate the region where >75% of the distortion was <2 mm. In the sagittal plane, the ROI dimensions were 330.7±13.7 mm (foot–head) and 292.3±6.2 mm (ant–post). In the transverse plane, the ROI dimensions were 323.7±10.8 mm (left–right) and 288.8±8.6 mm (ant–post). Finally, in the coronal plane, the ROI dimensions were 286.1±16.1 mm (foot–head) and 335.6±10.4 mm (left–right). Figure 6 demonstrates the results of the 3D distortion characterization obtained via DIR between CT‐SIM and MR‐SIM images. Distortions at the edges of the FOV can be observed in the top row of Fig. 6. Once DIR was applied, the agreement in the marker alignment improved substantially. Figure 3(d) summarizes the distortion analysis via the DVFs for varied radii from isocenter. Within a 5 cm radius of isocenter, the mean distortion was 0.59±0.32 mm (maximum=1.65 mm). For 5–10 cm from isocenter, the mean distortion was 0.88±0.51 mm (maximum=3.05 mm). However, farther from isocenter (10–15 cm), distortions were larger (mean=1.57±1.06 mm, maximum=6.26 mm) and for 15–20 cm from isocenter, distortions were 2.37±1.41 mm (maximum=7.33 mm). Within a 10 cm radius of isocenter, only 3.8% of the voxels yielded distortions ranging from 2–3 mm. However, at greater distances from the isocenter, 24.3% of the voxels within the 15–20 cm annulus distorted >3 mm.

### E. Laser quality assurance

#### E.1 Isocenter offset and alignment

Periodic laser motion and alignment tests were all within AAPM TG‐66 recommendations (ELPS<2 mm deviation across scan plane, absolute laser movement <1 mm from expected) over the timeframe tested.

#### E.2 Laser impact on image quality

Clear patterns of interference were manifested when the ELPS was turned on during the spurious noise test. The discrete noise frequencies are within the operating frequency band of the MR scanner (not shown). The induced noise exhibited as line patterns rather than as a general increase in the background noise. When the PIQT image quality tests were run with the lasers on, the signal‐to‐noise ratio test failed (reduction of 4.6%−12.6% for TE values of 50−150 ms).

### F. Quality assurance results

A slight reduction in central frequency was measured over time, with the largest day‐to‐day variation of 42 Hz, which was within the ACR recommendations of <1.5 ppm daily change^(19)^ (~64 Hz based on our initial acceptance levels). At ~120 days postacceptance, a large reduction (276 Hz from previous measurement) in central frequency was observed. At day 106, the MR‐SIM unit underwent service, and afterward, the new central frequency (42,587,276 Hz) was still within manufacturer specifications (42,584,000−42,591,000 Hz). According to the ACR recommendations, a service‐related change in center frequency can be accepted provided it can be explained, and the center frequency action criteria of <1.5 ppm can be applied to this new baseline.^(19)^ Transmitter gain was stable (0.63±0.01 dB, range: 0.62−0.64 dB) and slice thickness accuracy was also consistent (FWHM=4.94±0.03 mm, nominal slice width=5 mm). Little deviation was observed for low contrast detectability, as measured via the SNR measurement for the head coil.

### G. Initial patient experience

#### G.1 Immobilization devices

MR compatible devices in primary use for our scanner include indexing bars, sponges, Alpha cradles (Smithers Medical Products Inc., North Canton, OH), Aquaplast masks (Aquaplast Inc., Avondale, PA), plastic headrests, rubber bands for banding feet, and BlueBAG BodyFIX Vacuum Cushions (Elekta, Stockholm, Sweden;vacuum pump not compatible) as listed in Table 1.

#### G.2 UTE/Dixon results

**Figure 7:**
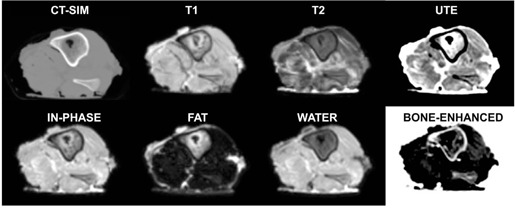
Axial CT‐SIM and 1.0T MR‐SIM images for a porcine shank, including ultrashort echotime (UTE), water and fat images generated via Dixon acquistions, and a bone‐enhanced image derived from the in‐phase and UTE datasets.

**Figure 8:**
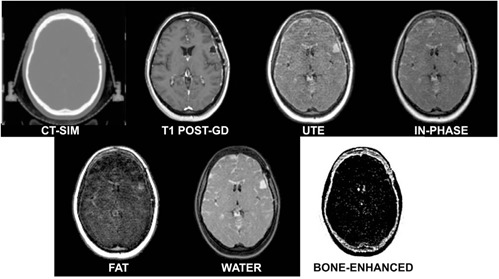
Axial CT‐SIM and 1.0T MR‐SIM images for a postsurgical stereotactic radiosurgery patient with a left frontal metastasis, including T1 post‐Gd, ultrashort echotime (UTE), water and fat images generated via Dixon acquisitions, and a bone‐enhanced image derived from the in‐phase and UTE datasets.

Figure 7 summarizes the CT and MR results for the porcine shank. The fat and water images were generated by the Dixon sequences, and the bone‐enhanced dataset was generated via postprocessing. Note the enhancement of the cortical bone in the UTE image and derived bone‐enhanced image. Figure 8 demonstrates the UTE/Dixon results for the radiosurgery brain case. Note that the bone signal is very bright on the CT scan, and there is little to no signal on the corresponding T1‐Gd, water, or fat Dixon images. On the UTE and in‐phase images, however, bone signal can be observed. Further image manipulation to highlight the bony features on UTE revealed strong bone enhancement. It can be noted that some signal in the left lateral ventricle was also highlighted as bone. The high‐intensity “halo” signal removed via postprocessing was likely caused by the radial acquisition technique. Ideally, the radial spokes (i.e., k‐space lines) should start from the center of k‐space and end on the surface of a sphere, but due to gradient delay, an offset exists between the start of the spokes and the center of k‐space. This “halo effect” can be mitigated by optimizing the selection of the gradient delay (or the trajectory delay indicated on our scanner), which is currently under evaluation at our institution. Nevertheless, the clear bone enhancement with UTE compared to other MR sequences is promising for future image segmentation work.

#### G.3 Four‐dimensional MRI (4D MRI)

Figure 4 (bottom) summarizes a phantom 4D MRI experiment separated into 10 breathing phases. Note the excursion of the square signal generator with respect to the stationary circular bottle of signal generator, with a return to near the original location at 90% phase. Faster breathing periods had higher duty cycles than slow (50.4% for 3 s and 39.4% for 5 s, p<0.001). As expected, ~ fourfold acquisition time increase was measured for ten‐phase versus two‐phase. MIP renderings revealed that S‐I object extent was underestimated a maximum of 8% (6 mm) for two‐phase 4D MRI as compared to ten‐phase. Acquiring 4 to 10 phases yielded nearly equivalent object excursion and volume (<2% from 10‐phase).

**Figure 9:**
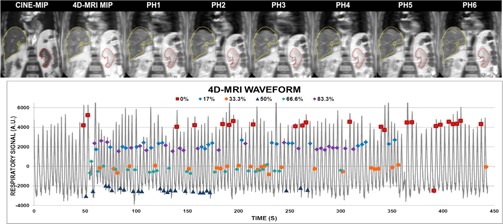
(Top) Coronal view for six‐phase 4D MRI for a liver cancer patient acquired in ~7.4 min. Contours derived from CINE‐MIP. (Bottom) 4D MRI waveform with respiratory tagging shown.

Figure 9 shows results for 6‐phase liver cancer 4D MRI, showing good image quality and no sorting artifacts. The regions of interest were derived on the 4D MRI MIP and overlaid to illustrate liver excursion. The nominal scan time was ~220 s, while the total acquisition time was 440 s, not including the training phase, yielding a duty cycle of ~50%. The overall exam time was within clinically acceptable limits.

## IV. DISCUSSION

This work sought to describe the initial clinical experience with a dedicated 1.0T MR‐SIM, including description of clinical workflow, MR‐SIM characterization, establishment of quality assurance procedures, and initial patient results. Templates for daily QA procedures that we have developed at our institution were provided to assist others embarking on MR‐SIM integration in radiation oncology. Laser QA was conducted as outlined by TG‐66 for CT‐SIM QA, and results were within the specifications (<2 mm for daily QA, <2 mm deviation with respect to laser scribes with couch motion, and individual laser motions <1 mm). These results are consistent to what was obtained by Mah et al.^(4)^ in their assessment of wall‐mounted lasers used in routine QA of a 0.23T MR‐SIM. While the lasers were shown to be stable, a need exists to interface the ELPS directly with the MR‐SIM console to enable marking isocenter on the MR‐SIM images, which will help facilitate MR‐only simulation.

All conducted image quality tests fell and were maintained within accepted criteria. Tracking central frequency was sensitive to a change due to machine service, and a slight reduction of central frequency over time was observed, as can be expected due to drifting postinstallation. The AAPM has suggested that one to two months postacceptance, the central frequency change is commonly <0.25 ppm/day,^(49)^ which will guide our new daily QA procedures.

To guide our clinicians on the expected accuracy due to image distortion at distances away from isocenter, 2D and 3D distortion analysis was conducted. Figure 6 showed that the largest displacements between the MR‐SIM and CT‐SIM occurred near the periphery, most notably in the anterior and posterior region for the axial view. This result was also observed in the 2D distortion analysis, where the 2 mm bounding box dimension of the anterior–posterior orientation was consistently smaller than the other dimensions. Because of the vertical field design, dedicated coils with integrated solenoid technology (Fig. 1(e) for the large body coil) are used with the receiver elements perpendicular to the body's long axis, thus reducing the anterior–posterior FOV. Our analysis revealed that distortions were negligible near scanner isocenter; however, they increased with increased distance from magnet isocenter (>2 mm in a 15–20 cm radius from isocenter). This suggests that efforts to correct for these distortions for large field of view acquisitions will be necessary to support MR‐SIM only treatment planning. It is important to note that the MR‐SIM scan of the 3D phantom (Fig. 3b) revealed a circular shutter, or mask, was present in the dataset, which could contribute to some DIR uncertainty, as we have shown that DIR has some inconsistencies at image boundaries.^(29)^

Wang et al.^(22)^ developed a $31\times 31\times 31\;{\text{cm}}^{3}$ phantom and an algorithm that includes >10,000 control points to evaluate 3D and correct for 3D distortion. The authors were able to reduce distortion‐induced errors from ~10 mm to less than 0.6 mm when their 3D correction was applied. In their follow‐up paper, 3D distortion of several MR scanners was characterized, revealing a strong dependence on distance to isocenter and manufacturer, with two scanners demonstrating increases in absolute maximum distortion of 2–3 mm for every 5 mm away from isocenter along the z‐axis.^(50)^ The 3D distortion phantom described in this work had coarser marker spacing (~2,500 control points) that are oriented cross‐sectionally in the axial plane and not in both planes simultaneously, which may limit some of the ability to conduct a full 3D analysis. Baldwin et al.^(51)^ used ~10,000 control points derived from a CT scan for their work, and Doran and colleagues^(27)^ used ~16,000 control points for their distortion characterization of a 1.5T scanner. However, these groups have performed their analyses using in‐house, large‐sized 3D distortion phantoms that they have developed for MRI. Unfortunately, the commercial availability of such large 3D distortion phantoms is currently quite limited, and this study was limited to a prototype phantom that was provided by the manufacturer. Despite these limitations, it is still informative to use the available equipment to characterize the 3D distortion that could be expected near the edges of the field of view. Furthermore, daily acquisition and quantitative analysis of 2D distortion for a large phantom in the transverse, sagittal, and coronal views has allowed for routine distortion assessment before daily clinical operation of the MR‐SIM.

This supports moving the object of interest toward the MR‐SIM isocenter to help mitigate this effect, and that distortion will most likely impact regions near the patient periphery. The phantom used in this work was $\sim 40\times 40\times 40\;{\text{cm}}^{3}$ and, due to its large size, the integrated quadrature coil was used. We expect that these results will be the worst‐case scenario. Another factor that contributes to geometrical distortion is magnetic field inhomogeneity originating from improper gradient offset adjustments or improper magnet shimming. It has been reported that open magnet fields have smaller volumes of gradient linearity that can contribute to dimensional errors in images,^(19)^ although this can be combatted by linking multiple patient positions together in the magnet's “sweet spots”.

Efforts to isolate different components of overall distortion due to system‐related distortions (i.e., magnetic field distortions and gradient nonlinearity) and patient/object‐induced distortions (i.e., chemical shifts and susceptibility) are currently underway.^(52)^ Susceptibility effects near interfaces, such as the sinuses and tissue, have been quantified by Wang et al.^(53)^ at 3.0T for the brain. They revealed that for a 3D‐T1 sequence, only 0.1% of the displacements were >2 mm; however, displacements of up to 4 mm were found. Sumanaweera et al.^(23)^ deduced that distortions at the bone/tissue boundary were negligible with respect to image resolution of ~1 mm. While the dosimetric impact of these distortions has yet to be fully characterized, it can be postulated that the largest dosimetric consequences would occur when a lesion was incorrectly identified, resulting in a geometric miss. The impact of using vendor provided system‐level distortion corrections was recently explored at 3.0T and revealed that displacements of up to ~4 mm were observed when vendor corrections were disabled.^(54)^ For a single‐fraction SRS treatment, six out of 18 displaced lesions studied had >20% of the target volume outside of the 90% isodose line. This suggests that proper management of all distortions, particularly in an MR‐only environment, is imperative. The phantom analysis we performed enabled assessment of the overall image quality of the MR‐SIM, although corrections for patient‐induced distortions cannot be managed by phantom evaluations and will need patient‐specific solutions.

The approach we are currently evaluating for a 4D MRI motion management solution was demonstrated using a programmed motion phantom experiment and a patient case. The underestimation of object volume using two‐phase 4D MRI acquisitions suggests that the acquisition of adequate phases (≥4) is important. This has also been a limitation described for 4D CT, where two‐phase internal target volumes (ITVs) underestimated ten‐phase ITVs by up to 12%.^(55)^ The described 4D MRI method builds upon methods previously proposed and validated by Hu et al.^(47)^ There are several other groups who have explored 4D MRI solutions, mostly simulating 4D CT reconstructions using 2D dynamic MRI acquisitions.^56^, ^57^, ^58^ Ideally, repetitive 3D volume acquisitions would be acquired to reconstruct real‐time 4D datasets, although it has been reported that frame rates can be quite low, yielding poor signal‐to‐noise ratio and possible interplay between anatomical motion and scan acquisition time.^(59)^ Four‐dimensional MRI results for a patient with liver cancer provided acceptable image quality and were acquired in ~7 min. Image contrast was appropriate for delineation of the liver and kidney, although further validation against 4D CT is warranted.

It should be noted that scan times are much longer for MR‐SIM than CT‐SIM, with a single MR scan taking 4–7 min compared to <1 min for a CT scan. Our current MR‐SIM patient time slots are 1 hr long, with ~45 min of active scan time. This long scan time may complicate the management of physiological processes, such as bladder and rectal filling, during the MR‐SIM imaging session. However, gains in acquisition time have been obtained via parallel imaging, or the reconstruction of multichannel k‐space data sampled below the Nyquist sampling rate.^12^, ^60^ The acceleration factor from parallel imaging is related to the number of available coil elements, although factors of 2–3 are often used.^(61)^ Compressed sensing has also emerged as an attractive option to accelerate acquisition.^60^, ^62^ Other readily available methods to improve scan efficiency include increasing slice thickness or introducing a slice gap, although at the expense of through‐plane resolution.

Implementing a new technology such as MR‐SIM in radiation oncology requires a significant effort, including a close collaboration with our diagnostic radiology colleagues. Thus far, we have developed our QA processes and clinical workflow including immobilization devices and imaging sequences to integrate MR‐SIM into radiation oncology for prostate, spine, and brain cancer cases. In order to consider MR‐SIM as a single modality solution for treatment planning, several next steps are necessary. First, having MR‐SIM software that enables final marking of isocenter using MR images will be essential. Patient‐induced and system‐level distortion corrections must be implemented. We have developed a methodology for generating a synthetic CT from MRI data for dose calculation and revealed negligible (<1%) dose differences between the D99%, mean dose, and maximum dose to the clinical target volume of the prostate.^(13)^ MR‐DRR bounding box analysis yielded <0.6 mm difference in the anterior–posterior and lateral DRRs. In addition, we have performed rigid registration of ~400 cone‐beam CT (CBCT) to CT‐SIM and CBCT to MR‐SIM with an emphasis on the prostate/rectal interface for a cohort of ten prostate cancer patients.^(63)^ Differences in shift positions were <2 mm different in the anterior–posterior direction and <1 mm in the other two dimensions, thus demonstrating the agreement between the two simulation techniques for IGRT purposes. Future work will incorporate distortion corrections and extend the work to other treatment sites.

## V. CONCLUSIONS

Based on our clinical experience to date, we have integrated MR‐SIM into our radiation therapy workflow. Through a series of mechanical and image quality characterization tests, we have developed our routine quality assurance procedures necessary for further work involving MR‐guided radiation therapy planning.

## ACKNOWLEDGMENTS

Technical support provided by Melanie Traughber, Mo Kadbi, Mike Cavaliere, and Tim Nielsen from Philips Medical Systems is gratefully acknowledged. Philips Medical Systems is acknowledged for the use and transport of their 3D phantom for our evaluation. Denise Socia is acknowledged for her assistance in MR‐SIM. Henry Ford Health System holds research agreements with Philips Medical Systems. Work partially sponsored by Henry Ford Health System Internal Mentored Grant (Carri Glide‐Hurst).

## Supporting information

Supplementary Material

Supplementary Material

Supplementary Material

Supplementary Material

Supplementary Material

Supplementary Material

## Appendix A: Daily Quality Assurance Worksheet

**Table:**

*I. Safety*	*II. Periodic Image Quality Test*
*Daily Result*	*Daily Result*
Patient Alarm (Y/N)	Central Frequency (Hz)
RF Coils – No Visual Defects (Y/N)	Transmitter Gain (dB)
Intercom System (Y/N)	Spatial Resolution
Compressor (Y/N)	Artifact (Y/N)
Helium Upper (%)	Type
Helium Lower (%)	PIQT Report (no highlight)
Therapist Initials	

*III. ELPS Accuracy* (±2 mm *specification)*	
	*Offset (mm)*
Axial	
Sagittal	
Coronal	

*IV. 2D Geometric Distortion (Record 2 mm bounding box dimensions in each plane and box dimension.)*		
*Orientation*	*Axis*	*Dimension (mm)*
Transverse	Left‐Right Ant‐Post	
Sagittal	Foot‐Head Ant‐Post	
Coronal	Foot‐Head Left‐Right	
