# Supplementary figures and images for: Initial clinical experience with a radiation oncology dedicated open 1.0T MR‐simulation
Source: J Appl Clin Med Phys. 2015 Mar 8;16(2):218–40. doi: 10.1120/jacmp.v16i2.5201 (PMC5690096; doi:10.1120/jacmp.v16i2.5201)

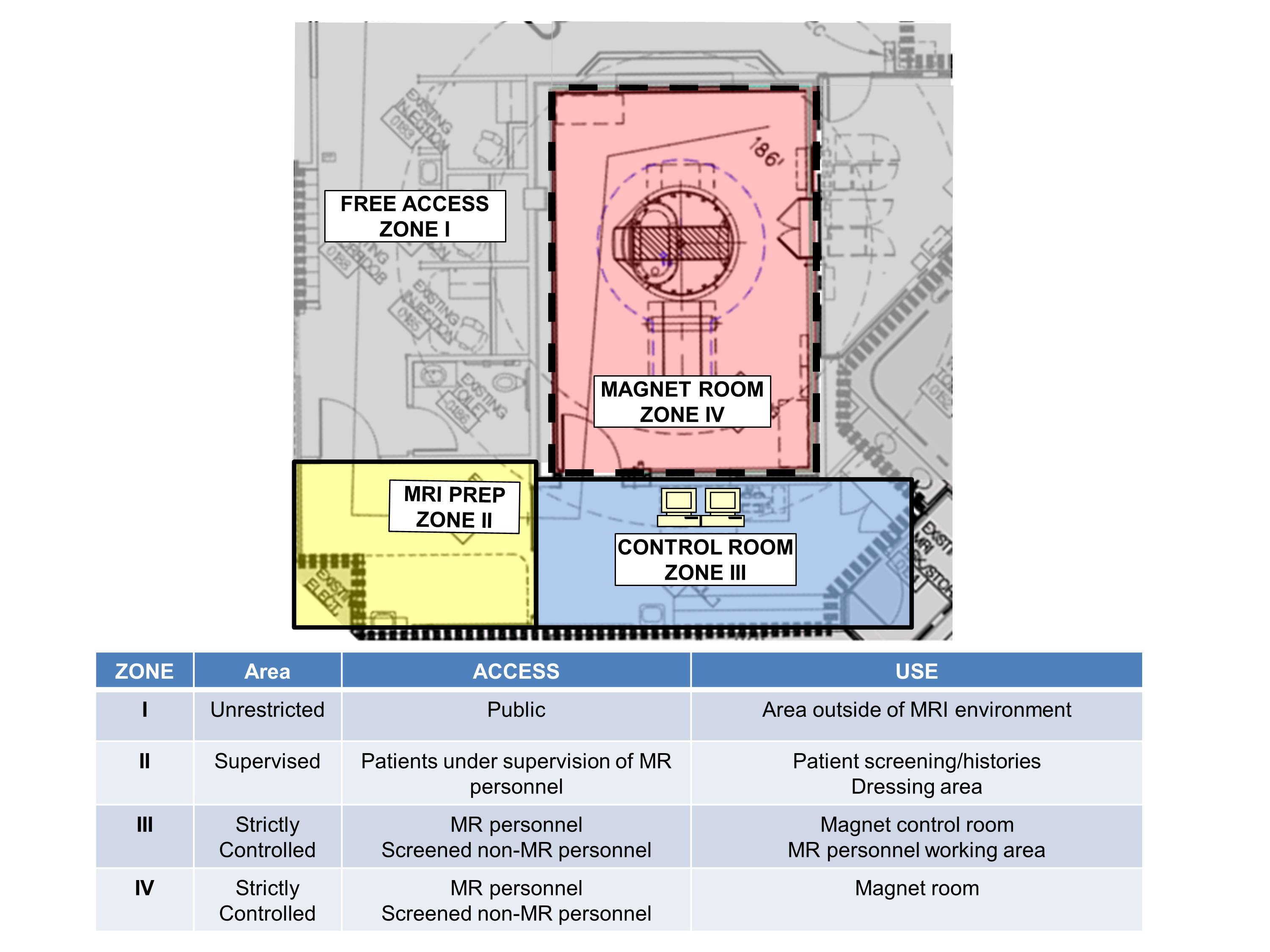

Supplement: Supplementary file 1 — Supplementary Material [file ACM2-16-218-s001.jpg]

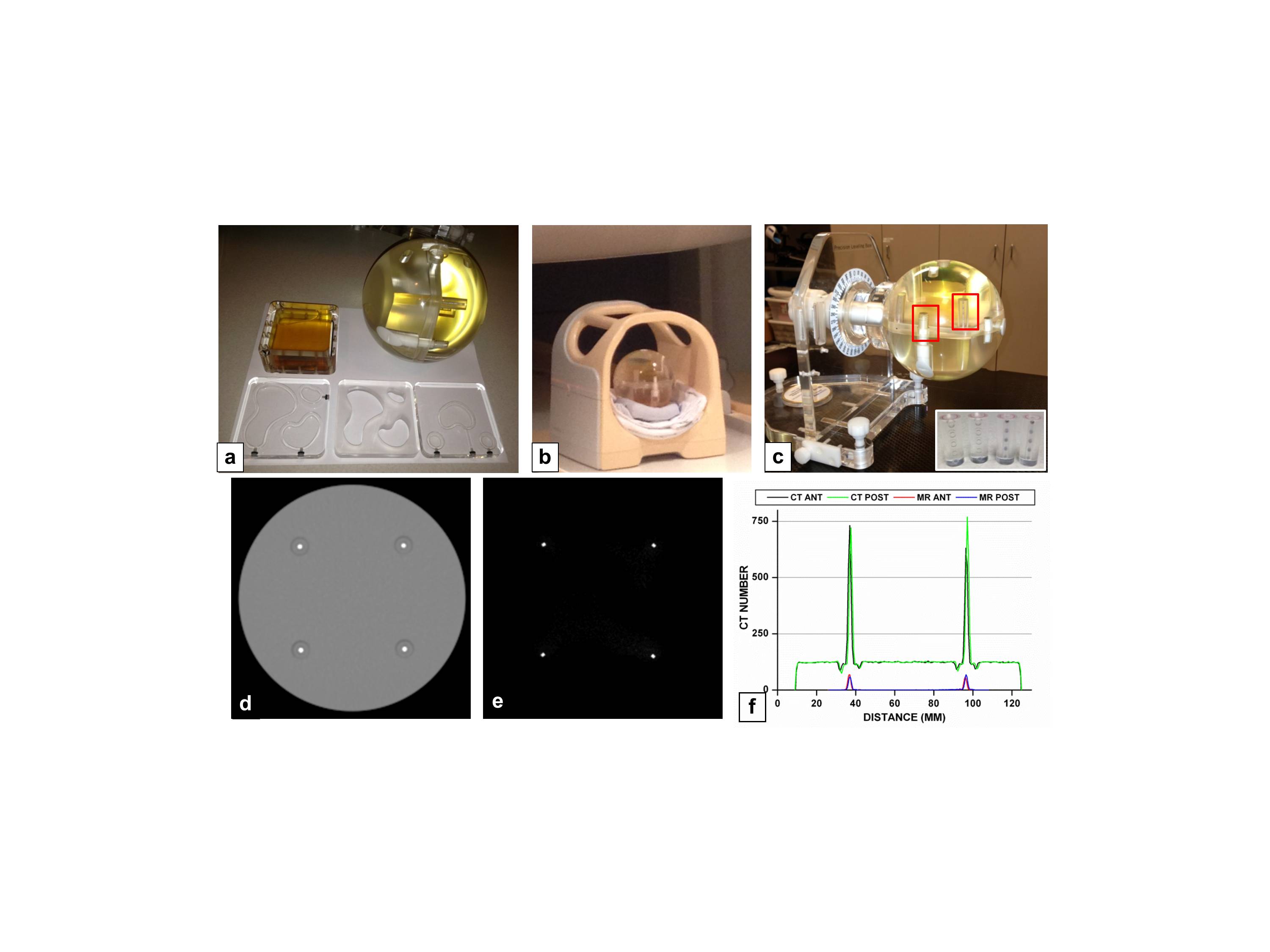

Supplement: Supplementary file 2 — Supplementary Material [file ACM2-16-218-s002.jpg]

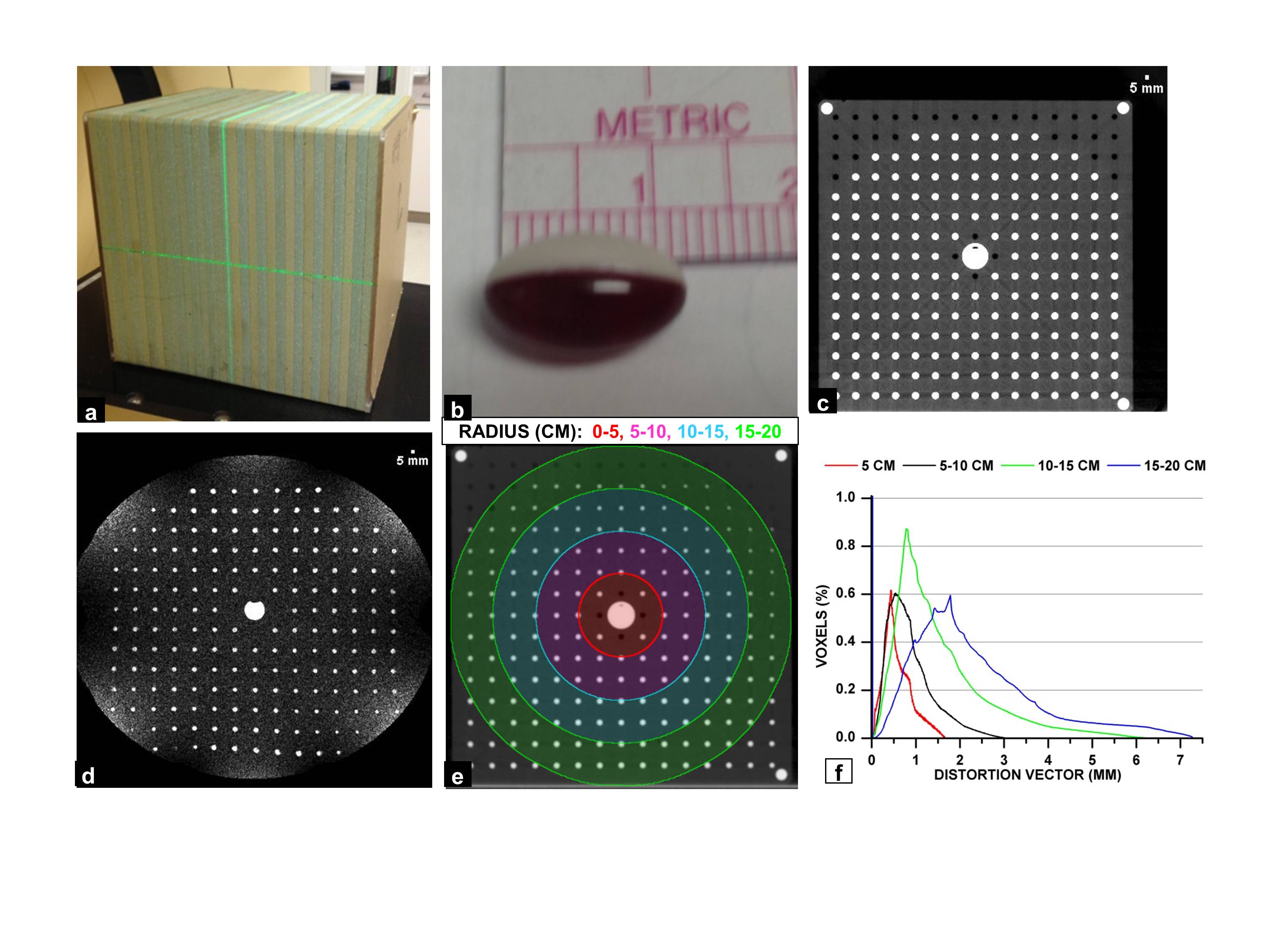

Supplement: Supplementary file 3 — Supplementary Material [file ACM2-16-218-s003.jpg]

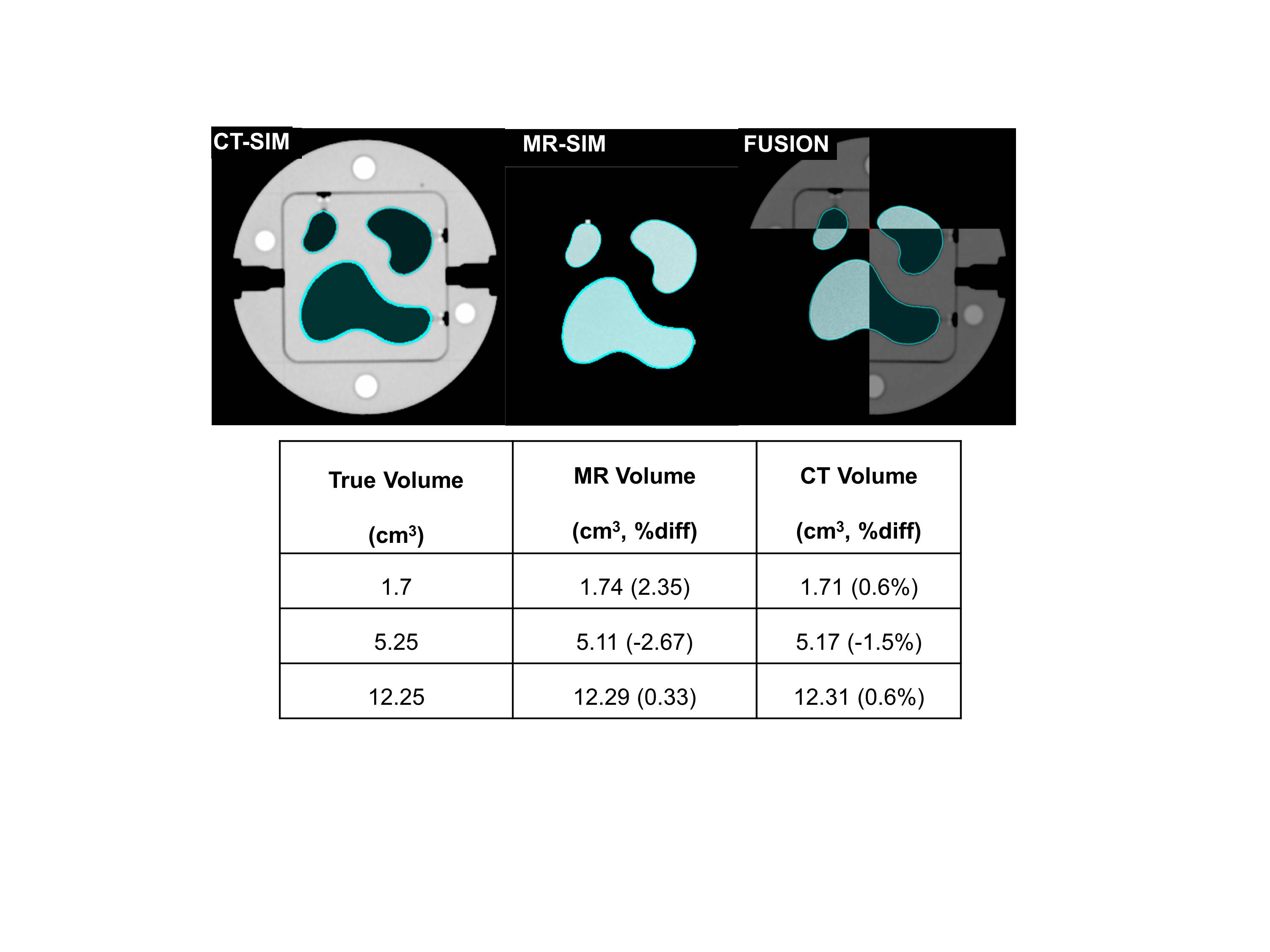

Supplement: Supplementary file 4 — Supplementary Material [file ACM2-16-218-s004.jpg]
