# Supplementary material for: Initial clinical experience with a radiation oncology dedicated open 1.0T MR‐simulation
Source: J Appl Clin Med Phys. 2015 Mar 8;16(2):218–40. doi: 10.1120/jacmp.v16i2.5201 (PMC5690096; doi:10.1120/jacmp.v16i2.5201)
Supplement: Supplementary file 5 — Supplementary Material [file ACM2-16-218-s005.docx]

Initial clinical experience with a radiation oncology dedicated open 1.0T MR-simulation

**Carri K. Glide-Hurst, Ning Wen, David Hearshen, Joshua Kim, Milan Pantelic,**

**Bo Zhao, Tina Mancell, Kenneth Levin, Benjamin Movsas, Indrin J. Chetty,**

**M. Salim Siddiqui**

*Department of Radiation Oncology*

*Henry Ford Health System*

*2799 West Grand Boulevard, Detroit, Michigan 48202*

Corresponding author: Carri Glide-Hurst, PhD

*churst2@hfhs.org*

Running Title: 1.0T MR-SIM for Radiation Oncology

Initial clinical experience with a radiation oncology dedicated open 1.0T MR-simulation

**Abstract:** Purpose: To describe our experience with 1.0T MR-SIM including characterization, quality assurance (QA) program, and features necessary for treatment planning.

Methods: Staffing, safety, and patient screening procedures were developed. Utilization of an external laser positioning system (ELPS) and MR-compatible couchtop were illustrated. Spatial and volumetric analyses were conducted between CT-SIM and MR-SIM using a stereotactic QA phantom with known landmarks and volumes. Magnetic field inhomogeneity was determined using phase difference analysis. System-related in-plane distortion was evaluated and temporal changes were assessed. 3D distortion was characterized for regions of interest (ROIs) 5 - 20 cm away from isocenter. American College of Radiology (ACR) recommended tests and impact of ELPS on image quality were analyzed. Combined ultrashort echo-time DIXON (UTE-DIXON) sequence was evaluated. Amplitude-triggered 4D-MRI was implemented using a motion phantom (2-10 phases, ~2 cm excursion, 3-5s periods) and in a liver cancer patient. Duty cycle, acquisition time, and excursion were evaluated between maximum intensity projection (MIP) datasets.

Results: <2% difference from expected was obtained between CT-SIM and MR-SIM volumes, with a mean distance of <0.2 mm between landmarks. Magnetic field inhomogeneity was <2ppm. 2D distortion was <2mm over 28.6-33.6 mm of isocenter. Within 5 cm radius of isocenter, mean 3D geometric distortion was 0.59 ± 0.32 mm (maximum = 1.65 mm) and increased 10-15 cm from isocenter (mean = 1.57 ± 1.06 mm, maximum = 6.26 mm). ELPS interference was within the operating frequency of the scanner and was characterized by line patterns and a reduction in signal-to-noise ratio (4.6-12.6% for TE = 50-150 ms). Image quality checks were within ACR recommendations. UTE-DIXON sequences yielded detectability between bone and air. For 4D-MRI, faster breathing periods had higher duty cycles than slow (50.4% (3s) and 39.4% (5s), p<0.001) and ~4-fold acquisition time increase was measured for 10-phase versus 2-phase. Superior-inferior object extent was underestimated 8% (6mm) for 2-phase as compared to 10-phase MIPs, although <2% difference was obtained for ≥4 phases. 4D-MRI for a patient demonstrated acceptable image quality in ~7 minutes.

Conclusions: MR-SIM was integrated into our workflow and QA procedures were developed. Clinical applicability was demonstrated for 4D-MRI and UTE imaging to support MR-SIM for single modality treatment planning.

**KEYWORDS:** MR-simulation, quality assurance, MRI, distortion characterization

PACS: 87.56.Fc, 87.61.-c, 87.57.cp

**I. INTRODUCTION**

Historically, magnetic resonance imaging (MRI) has been integrated into radiotherapy treatment planning primarily as an adjunct to computed tomography (CT) to assist in tumor delineation for many treatment sites such as brain, liver, head and neck, and prostate. This conventional CT-based workflow relies on target and/or organ at risk (OAR) definition on the MRI, and a transfer of contours to CT via image registration for subsequent treatment planning. However, performing MRI to CT image registration introduces additional systematic uncertainties (typically ~ 2 mm) that can be detrimental to localization of target and organs at risk([1](#_ENREF_1), [2](#_ENREF_2)). Furthermore, having two separate simulations burdens the clinical workload and the use of CT-SIM exposes the patient to ionizing radiation. Thus, implementing MRI as a stand-alone simulation modality (i.e. MR-SIM) for radiation therapy treatment planning is advantageous. To this end, others have implemented radiation oncology dedicated MR scanners, including characterization of low-field MR, and integration of data into treatment planning for delineation and dose calculation([3-5](#_ENREF_3)). For example, Mah *et al.* described their implementation of 0.23T MR-SIM, including external lasers used for virtual isocenter and distortion quantification and correction([4](#_ENREF_4)). Kapanen *et al.* described their commissioning process for MRI-only treatment planning at 1.5T, although their work was focused specifically on implementation for prostate cancer([6](#_ENREF_6)). Integrated platforms between the MRI and linear accelerator, often incorporating patient trolley systems to assist with similar setup between imaging and treatment, have also been described([7-9](#_ENREF_7)).

Recently, dedicated MR-SIM platforms have been introduced, adding features to further improve integration into radiation therapy such as flat table tops, external laser systems that interface with scanner software, and dedicated radiation therapy imaging protocols. Here, we describe our initial experience with one of the only radiation-therapy dedicated MR-SIM platforms clinically available([10](#_ENREF_10)). While other groups ([4](#_ENREF_4), [11-13](#_ENREF_11)) have reported on their implementation of different components of MRI as a simulator at other field strengths, we add to the literature by describing our initial clinical experience with a dedicated 1.0T open dedicated MR-SIM platform with a vertical magnetic field design. Specifically, we describe our personnel requirements and safety procedure, initial MR-SIM characterization including distortion quantification, establishment of a quality assurance (QA) program, immobilization devices, and specialized imaging sequences such as 4D-MRI and ultrashort echo time (UTE) and DIXON imaging for motion management and bone segmentation, respectively. This work characterizes one of the only commercially available radiotherapy-dedicated MR-SIM platforms currently available, and, coupled with ongoing work generating synthetic CTs([14](#_ENREF_14)) for dose calculation and the characterization of object-induced distortions, is a first step toward the overarching goal of using MR-SIM for single modality simulation at our institution.

**II. METHODS**

**II.A. Safety and Personnel Requirements for MR-SIM**

In the design of the MR-SIM suite, we followed the American College of Radiology (ACR) “four zone” concept -to restrict the areas affected by the magnetic field (i.e. Zones III (control room) and IV (magnet room))([15](#_ENREF_15)) as shown in Figure 1(left). Zone I is open to the general public and includes any area outside of the MRI environment. Zone II is our MRI preparation area used for MR screening, taking patient histories, and patient dressing. Zone II is the region in between the uncontrolled Zone I and strictly controlled Zones III and IV. Patients do not move from Zone II to Zone III unless under the supervision of Level 2 MR personnel or those who have been trained to ensure MR safety guidelines are strictly adhered to for patient, personnel, and equipment safety([15](#_ENREF_15)). Our current Level 2 MR personnel consist of MR technologists, diagnostic medical physicists, and MR radiologists. By contrast, Level 1 personnel include those who have passed minimal safety educational requirements to ensure their safety as they work in Zone III, such as our radiation oncologists, therapy medical physicists, radiation oncology nursing staff, radiation therapists, and researchers. According to the ACR, while Level 1 personnel are allowed unaccompanied access to Zones III and IV, they *cannot* be responsible for non-MR personnel in Zone IV. Our MR-SIM team is led by: (1) a board-certified MR physicist who assisted with machine acceptance, quality assurance (QA), parameter optimization for clinical protocols, and MR safety training, and (2) a board-certified MR radiologist who provides qualitative feedback on image quality and acquisition sequences.

**II.B. Safety screening and patient workflow**

Efforts are necessary to develop and administer patient and personnel screening documentation and policies. In our institution, we adhere to existing policies developed by our Radiology Department that are similar to what has been outlined in the literature([15](#_ENREF_15)). Screening includes evaluating MR eligibility and the presence of contraindications, such as implanted metal objects/implants or non-removable body piercings, pregnancy, claustrophobia, and inability to lie still for >30 mins. The ACR recommends a minimum of two separate MR safety screening sessions conducted by two people, one of which is Level II MR personnel.([15](#_ENREF_15)) At our institution, patients are screened on three occasions: (1) initially, before the MR-SIM appointment has been scheduled to ensure eligibility, (2) the day of the MR-SIM, often in the Radiation Oncology department, and (3) in Zone II (i.e. before entering Zone III). We typically have our Radiation Oncology nursing staff conduct the initial screening, our radiation therapist conduct the second screening the day of the procedure, and the MR-technician conduct the final screening before the patient is scanned to ensure the most conservative approach to patient safety.

Due to limitations in the current version of the MR-SIM software (HFO RT Oncology Configuration, v3.5.2), we are unable to mark the final isocenter (set-up point) using MR images. This functionality, including the transfer of isocenter coordinates to the scanner and laser system for subsequent patient marking is expected in a future upgrade. Nevertheless, it is possible to use a reference point marking approach as described in TG-66([16](#_ENREF_16)), where MR-compatible skin markers and reference marks could be used. Given the current limitations in the software, our current workflow includes performing the CT-SIM and MR-SIM on the same day, with the CT-SIM preceding the MR-SIM appointment. We set isocenter and conduct patient marking using CT-SIM, and then transfer the isocenter coordinates to the MR-SIM LAP laser software (described in section II.C.2). The MR-SIM lasers will then move to the appropriate sagittal and lateral locations from the marked CT-SIM isocenter, and the immobilized patient can be positioned accordingly.  By importing the CT-SIM isocenter in this manner, patients can then be translated by a known physical offset to the magnet’s isocenter, thereby introducing the least amount of distortion in the anatomy of interest. With added functionality, future work will involve exploring MR-SIM for patient marking.

An MR-SIM Time Out checklist is also administered before the beginning of scanning to ensure patient safety. The Time Out checklist is based on the “Universal Protocol” recommended by the *Joint Commission* to prevent surgical errors([17](#_ENREF_17)) and has been adapted for our imaging and radiation therapy process to ensure proper patient identity, treatment site, MRI safety screenings, patient positioning, presence of two hearing protection devices (i.e. ear plugs and headphones), and absence of skin folds and patient loops (i.e. skin to skin contact in hands touching)and that the lasers are turned off (see section II.H.2.b) before scanning procedures are initiated.

**II. C. MR-Simulator and auxiliary components**

**II.C.1 MR-Simulator and software**

The 1.0 T Philips Panorama High Field Open (HFO) Magnetic Resonance System (Philips Medical Systems, Cleveland, OH) (MR-SIM) was installed in January, 2013 and acceptance testing was performed in March, 2013. The system consists of a vertical magnetic field design with 160 cm wide aperture and a 45 cm field of view (Figure 1(right)), which enables lateral table translation of up to 28 cm to center the anatomy of interest at the magnet isocenter. Because of the vertical field design, dedicated coils with integrated solenoid technology (Figure 1(e) for the large body coil) are used with the receiver elements perpendicular to the body’s long axis. Another benefit of the rigid coils is that many MR-compatible immobilization devices can be accommodated while maintaining the patient’s external contour for treatment planning purposes.

The MR-SIM has integrated software that includes QA imaging sequences (distortion correction, laser QA), functionality to swap the laser system (described in detail later), and initial radiation therapy Exam Cards (i.e. pre-programmed image acquisition settings) for brain, pelvis, head and neck, and gynecological cases.

**II.C.2 Laser systems**

The MR-SIM consists of two laser systems: an integrated laser (Fig 1(d), also called the “light visor”) and MR-compatible external laser positioning system (ELPS) (Fig 1(b)). The integrated light visor laser is a Class II, 635 nm laser at the end of the bore used to select the plane that will be positioned in the center of the magnet and to assist with patient positioning. The ELPS DORADOnova MR3T (LAP of America Laser Applications, Boynton Beach, FL) consists of 6 Class II sagittal, transverse, and coronal external lasers used for patient positioning and translational/rotational alignment. The added external lasers are to enable correlation between external skin marks and MR images. The ELPS is similar to the CT-SIM laser system where the patient is localized using a virtual isocenter outside of the magnet bore([16](#_ENREF_16)), although the MR-SIM software does not currently allow for interactive isocenter marking using patient images. The MR console is configured to use the ELPS system for patient positioning, although this communication must be manually enabled on a daily basis. If the patient has undergone a CT-SIM with isocenter marking, the isocenter coordinate information can be imported from CT-SIM into the ELPS software and loaded. The lasers will then move to the appropriate sagittal and lateral locations from the marked isocenter, and the patient can be positioned accordingly. It is important to note that while the patient support system trolley can move vertically, the couch does not adjust in the vertical direction once it is inside the bore. Finally, while patients can be localized using the ELPS, the table will be laterally translated to move the anatomy of interest to the isocenter, which is particularly important for lateral lesions such as breast or extremities. During patient scanning, the ELPS must be disabled, as it degrades image quality as evaluated in Section II.H.2.b.

**II.C.3 MR-compatible couchtops**

The Indexed Patient Positioning System (IPPS™ Overlay, CIVCO Medical Solutions, Kalona, Iowa) is a flat couch overlay device with indexing capability with two different sizes: 40 cm width for upper body imaging, and 50 cm width for pelvic/lower body imaging. An MR-compatible Lok-Bar (CIVCO Medical Solutions, Kalona, Iowa) consists of three pins to enable consistent positioning of immobilization devices between MR-SIM and CT-SIM/radiation therapy. As shown in Fig 1(f), the couchtop sits on the patient support system on top of risers that allow the couch to be 18.5 mm above the MR-SIM patient support system and coils, thereby preventing the coils from touching the patient or immobilization devices. The IPPS weight limit is 550 lbs (250 kg) and requires two personnel to affix it to the integrated patient support system.

**II.D. Volumetric analysis**

To compare volumes between CT-SIM and MR-SIM, the LUCY 3D Plus QA Phantom (Standard Imaging, Middleton, WI) (Fig 2(a)) was employed. The Lucy phantom is a high-precision, MR-compatible modular device that is specifically designed to meet the needs required for stereotactic QA([18](#_ENREF_18)). For both MR-SIM and CT-SIM acquisitions, the Lucy phantom was affixed to its precision leveling base (Fig 2(b)) Briefly, for MRI scanning, an MRI signal generator (i.e. cavity filled with manganese chloride solution) was first fitted into the Lucy phantom to produce appropriate levels of MR signal strength, as shown in Fig 2(a). An MRI volumetric insert containing three irregular shapes filled with mineral oil and known volumes was then imaged—these volumes were considered the “true volumes” based on the manufacturer-stated volume. MR-SIM scans were acquired with both a four element, phased-array head coil and a two element, phased-array Medium body/spine coil. Two frequently used clinical sequences were used: a 3D T1-weighted fast-field echo acquisition sequence (TE/TR/α = 6.9/25 ms/30º and pixel bandwidth = 112 Hz/pixel, and a 3D T2 turbo-spin echo sequence (TE/TR/α = 90/3683 ms/90º and pixel bandwidth = 150 Hz/pixel). Both sequences used the following parameters: FOV = 223 × 231 × 40 mm^3^, acquisition matrix = 332 × 323 mm^2^, and voxel size ≈ 0.69 × 0.69 × 1 mm^3^. For CT-SIM acquisition, a clinical stereotactic radiosurgery brain acquisition protocol was used with 1 mm axial slice thickness, 512×512 image dimension, 120 kVp, 284 mAs, and 0.68 × 0.68 mm pixel spacing.

All MR-SIM and CT-SIM phantom data were transferred to the Eclipse Treatment Planning System (Eclipse TPS, v11.0, Varian Medical Systems, Palo Alto, CA) for subsequent analysis. To standardize object contouring, small, medium, and large irregular regions of interest (ROIs) were segmented using a set window/level for each modality and automated thresholding in Eclipse as illustrated in Figure 2(f and g) for the CT-SIM and MR-SIM, respectively. Volume percent differences were calculated using known manufacturer-stated true volumes.

**II.E. Spatial fidelity and image registration**

Four marker cylinders, each containing five 2mm oil beads (MRI) or five 2mm aluminum beads (CT) (Figure 2(b)), fit into precision-machined cavities into one hemisphere of the Lucy 3D QA Phantom. One end of the cylinders has red etching for orientation purposes, and the oil beads and aluminum beads are spaced 5 mm center-to-center. The marker cylinders are oriented in a known, rigid geometry forming a rectangle of 60 mm center-to-center. Spatial fidelity was evaluated by generating line profiles across (right to left) the marker cylinders in the axial plane for the CT-SIM, T1-weighted, T2-weighted and head/body coil datasets, as shown in Figure 2(c-e). The peaks of the implanted cylinders were determined by finding the local maximum intensity for each marker via a bounding box and search function (OriginLab, version 6.1, OriginLab Corporation). Peak-picking was visually verified and modified to reflect the center of the peak if necessary. Absolute displacement and percent differences from expected were calculated for both CT-SIM and MR-SIM datasets for the four locations (anterior, posterior, left, and right, as shown in Figure 2(c)). MR-SIM to CT-SIM image registration was performed in the Eclipse Registration Workspace using automatic, intensity based image registration (rotation/translation) that employs a linear optimization method with mutual information calculation([19](#_ENREF_19)). The registration results between the CT and MRI marker cylinders were visually inspected for agreement.

**II.F. Initial magnetic field homogeneity characterization**

At time of acceptance, the initial magnetic field homogeneity was characterized using an implemented phase difference analysis technique recommended by the ACR([20](#_ENREF_20)). Briefly, a 31 cm diameter uniform spherical phantom was imaged with the quadrature body coil. 2D gradient echo images were acquired in axial, sagittal, and coronal planes using a large FOV covering the phantom with the following parameters: TE1/TE2/TR/α = 10/12/500 ms/20º, pixel bandwidth = 1420 Hz/pixel, acquisition matrix = 176 × 88 mm^2^, FOV = 350 mm^2^, voxel size ≈ 0.5 × 0.5 × 5 mm^3^. Phase difference maps were generated between the two TE datasets using in-house software to determine the pixel-by-pixel measurement of field homogeneity for each axis using the phase images. Images were normalized between 0 and 2π and transition zones (i.e. areas where the original phase variation exceeded 2π) were removed using a standard phase unwrapping algorithm([21](#_ENREF_21)). A phase difference map was generated by subtracting the phase image at TE = 10 ms from that at TE = 12 ms to yield phase difference values directly proportional to the magnetic field at each pixel([22](#_ENREF_22)).

**II.G. Geometric fidelity**

Geometric accuracy can be classified into two major components: system-related distortions (magnetic field distortions and gradient non-linearity) and patient/object-induced distortions (e.g. chemical shifts and susceptibility). Geometric distortion due to magnetic field inhomogeneity and gradient field non-linearity has been well-documented in the literature([23-29](#_ENREF_23)), although not specifically for the 1.0T HFO. 2D in-plane distortion is evaluated for routine daily QA using a vendor-provided geometric planar distortion phantom scanned in three planes (axial, sagittal, and coronal) using the integrated quadrature body coil. A vendor-supplied imaging protocol generates images over ~35 cm × 40 cm field of view (FOV) and 2D distortion is quantified using vendor-provided software. The software compares the acquired data to an ideal grid of phantom markers, and deviations between the expected and measured values based on center of mass analysis. 2D contours are then interpolated to the acquired images based on the calculated deviations, rendering a distortion plot with isocontours ranging from 2 to 6 mm for each plane. A rectangular ROI is automatically derived indicating the area where >75% of the distortion is <2 mm. The MR technician visually verifies that the location of the 2 mm isocontour does not extend substantially into the rectangular region, and records the dimensions of the rectangular ROI. In this manner, 2D distortion can be assessed on a routine basis, before the MR-SIM is used for daily operation, and temporal changes can be ascertained.

3D distortion characterization was performed during MR-SIM acceptance using a prototype phantom provided by the vendor. Because of the large phantom size (40 cm × 40 cm × 40 cm), the integrated quadrature body coil was used for scanning. The phantom consisted of Docusate Sodium capsules (~12 mm length, 6 mm diameter, Fig 3(a)) with 2.5 cm centroid-to-centroid in-plane spacing and 2.7 cm z-axis centroid-to-centroid spacing, yielding ~2500 control points over the phantom volume. The phantom was first scanned with CT-SIM (Philips Big Bore, Philips Medical Systems) with 600 mm^2^ FOV, 2 mm axial slice thickness, 120 kVp, 284 mAs, voxel size ≈ 1.19 × 1.19 mm^2^) as shown in Fig 3(a)). 3D T1 Fast-field echo acquisitions were acquired with the 1.0 T MR-SIM (TE*/*TR/α= 3.83/9 ms/10◦, pixel bandwidth = 191 Hz/pixel, voxel size ≈ 0.938 × 0.938 × 1 mm^3^, acquired using a FOV of 450 × 450 × 400 mm^3^) as shown in Figure 3(b).

To derive a 3D distortion map, deformable image registration (DIR) was conducted using Velocity Advanced Imaging (VelocityAI, v3.0.0, Velocity Medical Solutions, Atlanta, GA). We have previously benchmarked VelocityAI DIR using 11 computational models developed via finite element methods (FEM) from patient lung CT scans with simulated motion ranging from 1.8 cm to 3 cm([30](#_ENREF_30)). FEM-generated displacement vector fields (DVFs) served as the gold standard and mean errors were 1.0 ~ 3.0 mm for the computational phantoms, with regions of large displacements yielding larger registration errors. Extrapolating these results to much smaller distortion magnitudes expected in MR-SIM, <1 mm errors can be expected. Because the phantom was in slightly different positions between MR-SIM and CT-SIM, manual matching and automated rigid registration (1 pass) were performed. Then, deformable (1 pass) registration was implemented between the MR-SIM and CT-SIM data. With a perfect DIR, the deformed image should be exactly the same as the fixed image. Visual verification of the congruence of the MR-SIM and CT-SIM images was assessed through an overlay and checked via the split view, blending view and spyglass tools available in VelocityAI, which is consistent with our clinical practices([31](#_ENREF_31)). Image congruence was found to improve when the MR-SIM data was used as the primary dataset. When CT-SIM was used as the primary dataset, the larger FOV caused erroneous expansion of the MR-SIM volume at the image boundaries. In addition, the MR-SIM data showed some signal loss at the boundary (Fig 3(b)), which may also have affected the DIR performance.

DVFs between the MR-SIM and CT-SIM data were used to describe the vector distortion magnitude. Distortion histograms (i.e. percent volume versus vector distortion magnitude) for several ROIs around isocenter were derived (5 cm radius sphere, 5-10 cm radius annulus, 10-15 cm radius annulus, and 15-20 cm radius annulus, as shown in Fig 3(c and d)).

**II.H. Daily quality assurance procedures**

Daily quality assurance procedures are all conducted by the MR technician on days that the MR-SIM is in clinical or research use.

**II.H.1. Image quality assessment**

For ongoing daily quality assurance, we use a manufacturer-provided Plexiglas phantom (200 mm diameter, 110 mm length) containing fluid with known relaxation time accuracy of ±5%. Vendor supplied PIQT software is used that includes modules for all of the weekly ACR performance tests, including automated measures of table positioning accuracy, central (i.e. resonant) frequency, and transmitted gain. Image quality metrics are assessed using National Electrical Manufacturers Association (NEMA) standards that were developed to enable comparisons across other MR systems([32](#_ENREF_32), [33](#_ENREF_33)). Tests included spatial resolution, low contrast detectability, flood-field uniformity, and artifact detection (e.g. ghosting or lines). The PIQT also includes annual ACR QA recommendations, such as slice thickness accuracy (full-width half maximum (FWHM) of a 1 mm thick standard crossed-ramp phantom insert (~11º angle)) and integral uniformity. Results are monitored daily by the MR technician (see Appendix B) and on a monthly basis by the physicist performing monthly QA.

**II.H.2 Laser quality assurance**

**II.H.2.a Isocenter offset and alignment**

An offset between the external bridge lasers and the magnet isocenter is determined at time of commissioning and verified before daily use, in a manner analogous to CT-SIM. The LAP Aquarius MR phantom (Laser Alignment (LA phantom)) and three-point leveling platform for routine laser QA are used for daily laser QA. The phantom has copper sulfate signal generators and a 2mm thick, 15 cm long by 15 cm wide oil-filled internal crosshair that is imaged in each plane. Etched scribes on the external phantom surface coincide with the internal crosshair. The leveling platform affixes to the IPPS table and phantom grooves are aligned to the sagittal, transverse, and coronal external LAP lasers for translational and rotational alignment. The body coil is connected over the top of the phantom, and the phantom is automatically translated to the magnet isocenter inside the bore using the established offset. 24 images (8 in each orientation) are acquired using 2.0 mm slice thickness, 0 mm gap, and 200 mm field of view based on a vendor-supplied protocol. The offsets are then characterized by assessing the deviations of the internal crosshair from the origin in the anterior-posterior, right-left, and foot-head directions. A similar procedure is conducted to test the known longitudinal offset of the light visor laser (i.e. scanner’s integrated laser). Offsets of the internal crosshair from the implanted markers are recorded in all directions and are expected to be <2 mm.

**II.H.2.b Laser impact on image quality**

Because the ELPS is positioned inside the MR room, when powered on, the system produces radiofrequency interference that generates MR image artifacts. To characterize the impact on image quality, two separate tests were conducted: a spurious noise test and the PIQT test with and without the ELPS powered on. For the spurious noise test, a service protocol was run that isolates and detects other frequencies outside of the scanner. Images were acquired using the integrated quadrature body coil. For the PIQT test, image quality parameters were automatically evaluated using built-in clinical software, including flood field uniformity, SNR, spatial linearity, slice profile, and spatial resolution. It should be noted that evaluating SNR may not be appropriate for images containing known artifacts([34](#_ENREF_34)); however, it is instructive to verify that the established QA procedures will identify and prevent leaving the ELPS powered on during patient scanning. In addition, we have established a workflow in our Time Out procedure to ensure the ELPS has been powered off prior to patient scanning.

**II.I. Monthly quality assurance procedures**

**II.I.1. Image quality**

Our monthly QA procedures include scanning of a large ACR accreditation phantom to determine the minimum levels of performance for well-functioning MRI systems([35](#_ENREF_35)). The phantom contains seven image quality modules including: geometric accuracy, high-contrast spatial resolution, slice thickness and position accuracy, slice position accuracy, image intensity uniformity, percent signal ghosting, and low-contrast object detectability. Results are compared with those acquired at time of acceptance testing. We also monitor the central frequency over time.

**II.I.2. Laser motion and alignment**

We follow the recommendations provided by AAPM TG-66 for laser motion and alignment monthly QA([16](#_ENREF_16)). The ELPS laser movement should be accurate, linear and reproducible. The LA phantom is first set up and leveled. The phantom has machined grooves that are aligned to the ELPS transverse, sagittal and coronal lasers. To verify that the light visor laser and ELPS lasers are parallel and orthogonal to the scan plane, the couchtop is moved longitudinally and laterally while the lasers are observed to ensure deviations from the phantom grooves are less than +/-2 mm.

To verify the ELPS individual side laser movement, a calibrated ruler is taped to the side of the LA phantom. The vertical (Z) position of the side laser on the ruler is recorded. The laser is then moved by 200 mm using the in-room control monitor. The position is recorded and compared to the expected with a tolerance of 1 mm. The lasers are then sent back to the zero position, and the test is repeated for the other side laser. For the sagittal ELPS laser, the calibrated ruler is placed on the couchtop perpendicular to the sagittal laser. The sagittal laser is moved to +100mm and -100 mm, and the position of the laser on the ruler is recorded. The measured values should be within +/-1mm of expected values.

**II.J. Initial Patient Experience**

**II.J.1. Immobilization Devices**

One of the first tasks for patient scanning included identifying immobilization devices that are considered “MR Compatible” according to guidelines set forth by the Food and Drug Administration([36](#_ENREF_36)). Our MR-SIM patient experience has primarily included prostate, spine, and brain cases. Table 1 demonstrates the coils, immobilization devices, and typical MR-SIM sequences acquired. For lumbar spine, the exam time was quite long for 3D scanning and patient motion could sometimes compromise image quality. Thus, we have moved forward with 2D protocols for this treatment site to decrease scan time. In addition, we enable sensitivity encoding (SENSE factor of typically 1.3~2.0) whenever possible to shorten exam times and reduce motion artifacts. Typical exam times are 4-7 minutes per scan type, although they largely depend on the field of view selected. For thoracic spine, we are still refining our imaging techniques to acquire high quality images in the presence of respiratory motion.

All images obtained with the first 10 patients scanned with the MR-SIM were reviewed by a board-certified Radiologist (M.P.) for qualitative image assessment. Feedback was provided from the Radiologist and sequences were iterated and added as recommended.

**II.J.2. Ultrashort echo-time (UTE) and Dixon Techniques**

Historically, it has been difficult to discriminate between cortical bone and air in MRI images due to the extremely short T_2_ and T_2_* relaxation times and fast-decaying signals. One solution includes employing ultrashort echo-time (UTE) sequences to assist in cortical bone segmentation([37-39](#_ENREF_37)). Using UTE sequences to generate synthetic CTs for treatment planning purposes([40](#_ENREF_40), [41](#_ENREF_41)) or for combined PET/MRI units([42](#_ENREF_42), [43](#_ENREF_43)) has shown great promise. For tissue classification, such as distinguishing lipids (i.e. fat) from water, DIXON imaging has also proven useful([40](#_ENREF_40), [42](#_ENREF_42), [44-46](#_ENREF_44)). We have implemented a combined ultrashort echo-time (UTE) and DIXON (i.e. UTE-DIXON) image sequence on our 1.0T MR-SIM under an IRB-approved prospective imaging protocol. The novel 2-point DIXON has been shown to improve fat suppression, spatial resolution, and image quality in the abdomen compared to standard techniques([46](#_ENREF_46), [47](#_ENREF_47)). Advantages of combining DIXON and UTE into one image sequence include shortened exam time and reduced image registration uncertainties between datasets. The UTE-Dixon sequence includes a single triple-echo scan (one free-induction decay and two echoes for DIXON) acquired using a 3D center-out radial acquisition mode to enable the shortest TE time possible.

For initial implementation, 3D volumetric UTE-DIXON images of a porcine shank acquired with a 4 element phased-array head coil with the following parameters: TR = 8.14 ms, TE = 0.144 (UTE), 2.44, and 4.74 ms, flip angle = 25◦, and isotropic voxel size ≈1.6 × 1.6 × 1.6 mm^3^ and FOV = 230 × 230 × 230 mm^3^. The images are used to calculate water and fat components, with an overall scan time of ~5 minutes for the UTE-DIXON sequence. For comparison purposes, T1-weighted turbo-field echo (TR/TE= 7.46/3.69 ms, voxel size ≈ 0.94× 0.94 × 2.2 mm^3^) and T2-weighted (TR/TE = 3549/80 ms, flip angle = 90◦, voxel size ≈ 0.68× 0.68 × 3 mm^3^) were acquired. Using a similar experimental setup, a CT scan was also conducted using slice thickness of 2 mm and in-plane pixel size of 0.68 mm × 0.68 mm^3^ (512×512 matrix). All images were interpolated to match the UTE-DIXON image dimensions for comparison purposes. To further reduce the signal from longer T2 components, a “bone enhanced” image was generated using the Image Algebra post-processing package available on our scanner. This image was generated by scaling the UTE image by a factor of 1.3 and subtracting the in-phase image generated by DIXON to further highlight the bone region in the porcine shank. The scaling factor magnitude was determined ad hoc based on the optimal suppression of brain tissue while enhancing the bone. The bone-enhanced image exhibited a high intensity “halo” of signal around the shank that was removed via image post-processing (i.e. applying an image mask derived from the outer surface of the T1 dataset and setting the background to white to provide optimal contrast).

UTE-DIXON scans were conducted for a radiosurgery patient status post-resection of a left frontal solitary metastasis. The 4 element phased-array head coil and immobilization devices listed in Table 1 were used. 3D volumetric UTE-DIXON images were acquired using the same parameters as the porcine experiment. For comparison purposes, a post-Gadolinium enhanced T1-weighted turbo-field echo (TR/TE = 21/6.9 ms, voxel size ≈ 0.97× 0.97 × 2.0 mm^3^ with a FOV = 190 mm (right-left), 233 mm (ant-post), and 164 mm (foot-head) was also acquired. A CT scan was also conducted using slice thickness of 2 mm and in-plane pixel size of 0.68 mm × 0.68 mm^2^ (512×512 reconstruction matrix), and all images were resampled to match the UTE-DIXON image dimensions. A bone-enhanced image was also generated for the patient case in a manner similar to the porcine shank.

**II.J.3. Four-dimensional MRI (4D-MRI)**

**II.J.3.A. 4D-MRI Algorithm**

Efforts are currently underway to evaluate a respiratory-triggered, single-shot T2-weighted TSE 4D-MRI acquisition technique under a prospective IRB-approved protocol. The algorithm is a multi-slice 2D dynamic MRI acquisition that was first introduced by Hu *et al.* Image acquisition of different respiratory states for each MR slice is performed using amplitude-based triggered acquisition 4D-MRI described in detail in Reference 36. To ensure better image contrast (e.g. by allowing for longer TR times), data sampling is separated into multiple respiratory cycles to allow the magnetization to completely relax. During the 4D-MRI acquisition, an external waveform is derived from a clinically available air-filled cushion and pressure sensor tracking device. The waveform is initially tagged at the start of the magnet’s preparation state, and once the preparation state has concluded, a calibration period over the first 2 breathing cycles is analyzed to determine the trigger levels for the different respiratory phases with respect to the maximum and minimum respiratory signal levels obtained. Hu’s approach has been further refined to improve clinical efficiency by integrating proprietary measures to reduce acquisition time and stabilize triggering due to breathing anomalies.

**II.J.3.B. 4D-MRI Phantom Experiment and Initial Patient Result**

Phantom experiments were carried out using a programmable respiratory motion platform (ExacTrac Gating Phantom, Version 1.0, BrainLAB AG, Germany) with string attached that pulled a cube-shaped signal generator (and placed on a Lego™ trolley located inside the magnet room (experimental setup shown in Figure 4). The platform translated the signal generator in the superior-inferior (S-I) direction. A chest wall component moving simultaneously in the anterior-posterior direction was used to generate a respiratory waveform via a clinically available air-filled cushion and pressure sensor tracking device (Figure 4, left). For a stationary reference, a bottle of signal generator (1000 ml bottle of demineralized water/copper sulfate) was scanned in the same FOV. Programmed superior-inferior object excursion was ~3 cm using a sinusoidal waveform with both 3 and 5 second breathing cycles. It should be noted that the actual object excursion was reduced to ~1.5-2 cm due to the addition of the string and trolley system, which is similar to what has been reported in the literature([48](#_ENREF_48)). Two to ten phase T2-weighted TSE 4D-MRI images were acquired with TE*/*TR/α = 50.25/2000 ms/90◦, pixel bandwidth = 259 Hz/pixel, voxel size ≈0.98 × 0.98 × 5 mm, and FOV = 250 × 201 × 125 mm^3^. All MRI datasets were exported from the scanner in DICOM format. Maximum intensity projections (MIP) were derived using MATLAB® (Mathworks, Natick, MA) by calculating the maximum intensity values of all voxels throughout all 4D-MRI phases. Duty cycle (nominal programmed acquisition time divided by overall scan time) and excursion were evaluated between phase acquisitions.

A liver cancer patient was consented to a prospective IRB-approved protocol to optimize acquisition parameters for abdominal 4D-MRI (6 phases, TE*/*TR/α = 75/6100 ms/90◦, voxel ≈1×1×7 mm^3^). Duty cycle, scan time, and image quality were evaluated between phase acquisitions. For image quality comparison purposes, coronal cine-MRI (~1 frame/sec, ~50 seconds) was also acquired. MIP renderings were generated in a manner similar to the phantom experiment.

**III. RESULTS**

**III.A. Volumetric analysis**

CT-SIM volumes were <2% different from ground truth (maximum difference = -1.8% (0.03 cc) for the smallest insert). T1-and T2-weighted images acquired using the head coil, where the signal to noise ratio was appropriate for the size of the object being imaged, were within -1.3% and 2.1% of ground truth, respectively, for the larger inserts. Delineations of the small inserts were slightly larger than expected (1.2-2.4%, 0.02-0.04 cc). In the Medium body/spine coil, volumes were consistently smaller than ground truth (-1.7 to -2.7%) for the medium and large inserts. However, for the small insert, volumes were equal or slightly larger for the T2- and T1-weighted images, respectively. Overall, clinically acceptable results were obtained for both CT-SIM and MR-SIM (<2% difference from manufacturer-stated true volumes). The equivalent diameters were <1mm different for all CT-SIM and MR-SIM volumes.

**III.B. Spatial fidelity and image registration**

Figure 2(c) demonstrates the intensity profiles across the CT-SIM and MR-SIM cylindrical markers for the different acquisitions and coils. For CT-SIM, the mean distance between markers was 59.83 ± 0.02 mm (<0.3% difference from expected, 60 mm). For the T1-weighted and T2-weighted datasets, the mean distance between markers was 60.05 ± 0.19 mm and 59.99 ± 0.16, respectively, across both coils (<0.4% different from expected). When the head coil and Medium body coil were considered separately, the mean distances were 60.07 ± 0.19 and 59.97 ± 0.17 mm, respectively. The image fusion shown in Fig 2(h)(right) demonstrates the spatial concordance between the MR-SIM and CT-SIM images after rigid registration.

**III.C. Initial Magnetic Field Homogeneity Characterization**

Figure 5 demonstrates the magnetic field inhomogeneity maps of the MR-SIM. The volume root mean square was calculated to be 0.9 ppm over 31 cm diameter spherical volume, which was within ACR specifications. Due to the vertical magnet design, the coronal datasets show the most symmetrical distribution (i.e. left-right, anterior-posterior) because of the equidistance from the magnetic field.

**III.D. Geometric distortion**

2D distortion characterization was tracked temporally over the first 8 months of operation via a 2D distortion phantom and assessment of rectangular ROIs to indicate the region where >75% of the distortion was <2 mm. In the sagittal plane, the ROI dimensions were 330.7 ± 13.7 mm (foot-head) and 292.3 ± 6.2 mm (ant-post). In the transverse plane, the ROI dimensions were 323.7 ± 10.8 mm (left-right) and 288.8 ± 8.6 mm (ant-post). Finally, in the coronal plane, the ROI dimensions were 286.1 ± 16.1 mm (foot-head) and 335.6 ± 10.4 mm (left-right). Figure 6 demonstrates the results of the 3D distortion characterization obtained via DIR between CT-SIM and MR-SIM images. Distortions at the edges of the FOV can be observed in the top row of Figure 6. Once DIR was applied, the agreement in the marker alignment improved substantially. Figure 3(d) summarizes the distortion analysis via the DVFs for varied radii from isocenter. Within a 5 cm radius of isocenter, the mean distortion was 0.59 ± 0.32 mm (maximum = 1.65 mm). For 5-10 cm from isocenter, the mean distortion was 0.88 ± 0.51 mm (maximum = 3.05 mm). However, farther from isocenter (10-15 cm), distortions were larger (mean = 1.57 ± 1.06 mm, maximum = 6.26 mm) and for 15-20 cm from isocenter, distortions were 2.37 ± 1.41 mm (maximum = 7.33 mm). Within a 10 cm radius of isocenter, only 3.8% of the voxels yielded distortions ranging from 2-3 mm. However, at greater distances from the isocenter, 24.3% of the voxels within the 15-20 cm annulus distorted >3 mm.

**III.E. Laser quality assurance**

**III.E.1 Isocenter offset and alignment**

Periodic laser motion and alignment tests were all within AAPM TG-66 recommendations (ELPS <2 mm deviation across scan plane, absolute laser movement <1 mm from expected) over the timeframe tested.

**III.E.2. Laser impact on image quality**

Clear patterns of interference were manifested when the ELPS was turned on during the spurious noise test. The discrete noise frequencies are within the operating frequency band of the MR scanner (not shown). The induced noise exhibited as line patterns rather than as a general increase in the background noise. When the PIQT image quality tests were run with the lasers on, the signal-to-noise ratio test failed (reduction of 4.6-12.6% for TE values of 50-150 ms).

**III.F. Quality assurance results**

A slight reduction in central frequency was measured over time, with the largest day-to-day variation of 42 Hz, which was within the ACR recommendations of <1.5 ppm daily change([20](#_ENREF_20)) (~64 Hz based on our initial acceptance levels). At ~120 days post-acceptance, a large reduction (276 Hz from previous measurement) in central frequency was observed. At day 106, the MR-SIM unit underwent service, and afterward, the new central frequency (42,587,276 Hz) was still within manufacturer specifications (42,584,000-42,591,000 Hz). According to the ACR recommendations, a service-related change in center frequency can be accepted provided it can be explained, and the center frequency action criteria of <1.5 ppm can be applied to this new baseline([20](#_ENREF_20)). Transmitter gain was stable (0.63 ± 0.01 dB, range: 0.62-0.64 dB) and slice thickness accuracy was also consistent (FWHM = 4.94 ± 0.03 mm, nominal slice width = 5 mm). Little deviation was observed for low contrast detectability, as measured via the SNR measurement for the head coil.

**III.G. Initial Patient Experience**

**III.G.1. Immobilization devices**

MR Compatible devices in primary use for our scanner include indexing bars, sponges, Alpha cradles, Aquaplast masks, plastic headrests, rubber bands for banding feet, and BlueBAG™ BodyFIX Vacuum Cushions (Elekta, vacuum pump not compatible).

**III.G.2. UTE-DIXON Results**

Figure 7 summarizes the CT and MR results for the porcine shank. The fat and water images were generated by the DIXON sequences, and the bone-enhanced dataset was generated via postprocessing. Note the enhancement of the cortical bone in the UTE image and derived bone-enhanced image. Figure 8 demonstrates the UTE-DIXON results for the radiosurgery brain case. Note that the bone signal is very bright on the CT scan, and there is little to no signal on the corresponding T1-Gd, water, or fat DIXON images. On the UTE and in-phase images, however, bone signal can be observed. Further image manipulation to highlight the bony features on UTE revealed strong bone enhancement. It can be noted that some signal in the left lateral ventricle was also highlighted as bone. The high-intensity “halo” signal removed via post-processing was likely caused by the radial acquisition technique. Ideally, the radial spokes (i.e. k-space lines) should start from the center of k-space and end on the surface of a sphere, but due to gradient delay, an offset exists between the start of the spokes and the center of k-space. This “halo effect” can be mitigated by optimizing the selection of the gradient delay (or the trajectory delay indicated on our scanner), which is currently under evaluation at our institution. Nevertheless, the clear bone enhancement with UTE compared to other MR sequences is promising for future image segmentation work.

**III.G.3. Four-dimensional MRI (4D-MRI)**

Figure 4(bottom) summarizes a phantom 4D-MRI experiment separated into 10 breathing phases. Note the excursion of the square signal generator with respect to the stationary circular bottle of signal generator, with a return to near the original location at 90% phase. Faster breathing periods had higher duty cycles than slow (50.4% for 3s and 39.4% for 5s, p<0.001). As expected, ~4-fold acquisition time increase was measured for 10-phase versus 2-phase. MIP renderings revealed that S-I object extent was underestimated a maximum of 8% (6mm) for 2-phase 4D-MRI as compared to 10-phases (Fig.5, bottom). Acquiring 4 to 10 phases yielded nearly equivalent object excursion and volume (<2% from 10-phase).

Fig. 9 shows results for 6-phase liver cancer 4D-MRI, showing good image quality and no sorting artifacts. The regions of interest were derived on the 4D-MRI MIP and overlaid to illustrate liver excursion. The nominal scan time was ~220 seconds while the total acquisition time was 440 seconds, not including the training phase, yielding a duty cycle of ~50%. The overall exam time was within clinically acceptable limits.

**IV. DISCUSSION**

This work sought to describe the initial clinical experience with a dedicated 1.0T MR-SIM, including description of clinical workflow, MR-SIM characterization, establishment of quality assurance procedures, and initial patient results. Templates for daily QA procedures that we have developed at our institution were provided to assist others embarking on MR-SIM integration in Radiation Oncology. Laser QA was conducted as outlined by TG-66 for CT-SIM QA, and results were within the specifications (<2mm for daily QA, <2 mm deviation with respect to laser scribes with couch motion, and individual laser motions <1 mm). These results are consistent to what was obtained by Mah *et al* in their assessment of wall-mounted lasers used in routine QA of a 0.23T MR-SIM([4](#_ENREF_4)). While the lasers were shown to be stable, a need exists to interface the ELPS directly with the MR-SIM console to enable marking isocenter on the MR-SIM images, which will help facilitate MR-only simulation.

All conducted image quality tests fell and were maintained within accepted criteria. Tracking central frequency was sensitive to a change due to machine service, and a slight reduction of central frequency over time was observed, as can be expected due to drifting post-installation. The AAPM has suggested that 1-2 months post-acceptance, the central frequency change is commonly <0.25 ppm/day([49](#_ENREF_49)), which will guide our new daily QA procedures.

To guide our clinicians on the expected accuracy due to image distortion at distances away from isocenter, 2D and 3D distortion analysis was conducted. Figure 6 showed that the largest displacements between the MR-SIM and CT-SIM occurred near the periphery, most notably in the anterior and posterior region for the axial view. This result was also observed in the 2D distortion analysis, where the 2 mm bounding box dimension of the anterior-posterior orientation was consistently smaller than the other dimensions. Because of the vertical field design, dedicated coils with integrated solenoid technology (Figure 1(e) for the large body coil) are used with the receiver elements perpendicular to the body’s long axis, thus reducing the anterior-posterior FOV. Our analysis revealed that distortions were negligible near scanner isocenter, however they increased with increased distance from magnet isocenter (>2 mm in a 15-20 cm radius from isocenter). This suggests that efforts to correct for these distortions for large field of view acquisitions will be necessary to support MR-SIM only treatment planning. It is important to note that the MR-SIM scan of the 3D phantom (Figure 6) revealed a circular shutter, or mask, was present in the dataset, which could contribute to some DIR uncertainty as we have shown that DIR has some inconsistencies at image boundaries([30](#_ENREF_30)).

Wang *et al*. developed a 31 × 31 × 31 cm^3^ phantom and an algorithm that includes >10,000 control points to evaluate 3D and correct for 3D distortion([23](#_ENREF_23)). The authors were able to reduce distortion-induced errors from ~10 mm to less than 0.6mm when their 3D correction was applied. In their follow-up paper, 3D distortion of several MR scanners was characterized, revealing a strong dependence on distance to isocenter and manufacturer, with two scanners demonstrating increases in absolute maximum distortion of 2-3 mm for every 5 mm away from isocenter along the z-axis([50](#_ENREF_50)). The 3D distortion phantom described in this work had coarser marker spacing (~2,500 control points) that are oriented cross-sectional in the axial plane and not in both planes simultaneously, which may limit some of the ability to conduct a full 3D analysis. Baldwin *et al.* used ~10,000 control points derived from a CT scan for their work([51](#_ENREF_51)), and Doran and colleagues used ~16,000 control points for their distortion characterization of a 1.5T scanner([28](#_ENREF_28)). However, these groups have performed their analyses using in-house large-sized 3D distortion phantoms that they have developed for MRI. Unfortunately, the commercial availability of such large 3D distortion phantoms is currently quite limited, and this study was limited to a prototype phantom that was provided by the manufacturer. Despite these limitations, it is still informative to use the available equipment to characterize the 3D distortion that could be expected near the edges of the field of view. Furthermore, daily acquisition and quantitative analysis of 2D distortion for a large phantom in the transverse, sagittal, and coronal views has allowed for routine distortion assessment before daily clinical operation of the MR-SIM.

This supports moving the object of interest toward the MR-SIM isocenter to help mitigate this effect, and that distortion will most likely impact regions near the patient periphery. The phantom used in this work was ~40 × 40 × 40 cm^3^, and due to its large size, the integrated quadrature coil was used. We expect that these results will be the worst-case scenario, Another factor that contributes to geometrical distortion is magnetic field inhomogeneity originating from improper gradient offset adjustments or improper magnet shimming. It has been reported that open magnet fields have smaller volumes of gradient linearity that can contribute to dimensional errors in images([20](#_ENREF_20)), although this can be combatted by linking multiple patient positions together in the magnet’s “sweet spots”.

Efforts to isolate different components of overall distortion due to system-related distortions (i.e. magnetic field distortions and gradient non-linearity) and patient/object-induced distortions (i.e. chemical shifts and susceptibility) are currently underway ([52](#_ENREF_52)). Susceptibility effects near interfaces, such as the sinuses and tissue, have been quantified by Wang *et al.* at 3.0T for the brain. They revealed that for a 3D-T1 sequence, only 0.1% of the displacements were >2mm, however displacements of up to 4 mm were found([53](#_ENREF_53)). Sumanaweera *et al.* deduced that distortions at the bone/tissue boundary were negligible with respect to image resolution of ~1 mm([24](#_ENREF_24)). While the dosimetric impact of these distortions has yet to be fully characterized, it can be postulated that the largest dosimetric consequences would occur when a lesion was incorrectly identified, resulting in a geometric miss. The impact of using vendor provided system-level distortion corrections was recently explored at 3.0T and revealed that displacements of up to ~4 mm were observed when vendor corrections were disabled([54](#_ENREF_54)). For a single fraction SRS treatment, six out of 18 displaced lesions studied had > 20% of the target volume outside of the 90% isodose line. This suggests that proper management of all distortions, particularly in an MR-only environment, is imperative. The phantom analysis we performed enabled assessment of the overall image quality of the MR-SIM, although corrections for patient-induced distortions cannot be managed by phantom evaluations and will need patient-specific solutions.

The approach we are currently evaluating for a 4D-MRI motion management solution was demonstrated using a programmed motion phantom experiment and a patient case. The underestimation of object volume using 2-phase 4D-MRI acquisitions suggests that the acquisition of adequate phases (≥4) is important. This has also been a limitation described for 4DCT, where two-phase internal target volumes (ITVs) underestimated ten-phase ITVs by up to 12%([55](#_ENREF_55)). The described 4D-MRI method builds upon methods previously proposed and validated by Hu *et al(*[*56*](#_ENREF_56)*).* There are several other groups who have explored 4D-MRI solutions, mostly simulating 4DCT reconstructions using 2D dynamic MRI acquisitions([57-59](#_ENREF_57)). Ideally, repetitive 3D volume acquisitions would be acquired to reconstruct real-time 4D datasets, although it has been reported that frame rates can be quite low, yielding poor signal-to-noise ratio and possible interplay between anatomical motion and scan acquisition time([60](#_ENREF_60)). 4DMRI results for a patient with liver cancer provided acceptable image quality and were acquired in ~7 minutes. Image contrast was appropriate for delineation of the liver and kidney, although further validation against 4DCT is warranted.

It should be noted that scan times are much longer for MR-SIM than CT-SIM, with a single MR scan taking 4-7 minutes compared to <1 minute for a CT scan. Our current MR-SIM patient time slots are 1 hour long, with ~45 minutes of active scan time. This long scan time may complicate the management of physiological processes such as bladder and rectal filling during the MR-SIM imaging session. However, gains in acquisition time have been obtained via parallel imaging, or the reconstruction of multi-channel k-space data sampled below the Nyquist sampling rate([13](#_ENREF_13), [61](#_ENREF_61)). The acceleration factor from parallel imaging is related to the number of available coil elements, although factors of 2-3 are often used([62](#_ENREF_62)). Compressed sensing has also emerged as an attractive option to accelerate acquisition ([61](#_ENREF_61), [63](#_ENREF_63)). Other readily available methods to improve scan efficiency include increasing slice thickness or introducing a slice gap, although at the expense of through-plane resolution.

Implementing a new technology such as MR-SIM in Radiation Oncology requires a significant effort, including a close collaboration with our diagnostic radiology colleagues. Thus far, we have developed our QA processes and clinical workflow including immobilization devices and imaging sequences to integrate MR-SIM into radiation oncology for prostate, spine, and brain cancer cases. In order to consider MR-SIM as a single modality solution for treatment planning, several next steps are necessary. First, having MR-SIM software that enables final marking of isocenter using MR images will be essential. Patient-induced and system-level distortion corrections must be implemented. We have developed a methodology for generating a synthetic CT from MRI data for dose calculation and revealed negligible (<1%) dose differences between the D99%, mean dose, and maximum dose to the clinical target volume of the prostate([14](#_ENREF_14)). MR-DRR bounding box analysis yielded <0.6 mm difference in the anterior-posterior and lateral DRRs. In addition, we have performed rigid registration of ~400 cone-beam CT (CBCT) to CT-SIM and CBCT to MR-SIM with an emphasis on the prostate/rectal interface for a cohort of 10 prostate cancer patients([64](#_ENREF_64)). Differences in shift positions were <2 mm different in the anterior-posterior direction and <1 mm in the other two dimensions, thus demonstrating the agreement between the two simulation techniques for IGRT purposes. Future work will incorporate distortion corrections and extend the work to other treatment sites.

**V. CONCLUSION**

Based on our clinical experience to date, we have integrated MR-SIM into our radiation therapy workflow. Through a series of mechanical and image quality characterization tests, we have developed our routine quality assurance procedures necessary for further work involving MR-guided radiation therapy planning.

**ACKNOWLEDGEMENTS**

Technical support provided by Melanie Traughber, Mo Kadbi, Mike Cavaliere, and Tim Nielsen from Philips Medical Systems are gratefully acknowledged. Philips Medical Systems is acknowledged for the use and transport of their 3D phantom for our evaluation. Denise Socia is acknowledged for her assistance in MR-SIM.

**Conflict of Interest:** Submitting institution holds a research agreement with Philips Medical Systems. Work partially sponsored by (Redacted) Grant (Author).

**References**

1. Roberson PL, McLaughlin PW, Narayana V, Troyer S, Hixson GV, Kessler ML. Use and uncertainties of mutual information for computed tomography/magnetic resonance (CT/MR) registration post permanent implant of the prostate. Med Phys. 2005;32(2):473-82. doi: doi:<http://dx.doi.org/10.1118/1.1851920>.

2. Dubois DF, Bice WS, Prestige BR. CT and MRI derived source localization error in a custom prostate phantom using automated image coregistration. Med Phys. 2001;28(11):2280-4. doi: doi:<http://dx.doi.org/10.1118/1.1406525>.

3. Krempien RC, Schubert K, Zierhut D, Steckner MC, Treiber M, Harms W, et al. Open low-field magnetic resonance imaging in radiation therapy treatment planning. International Journal of Radiation Oncology*Biology*Physics. 2002;53(5):1350-60. doi: <http://dx.doi.org/10.1016/S0360-3016(02)02886-9>.

4. Mah D, Steckner M, Palacio E, Mitra R, Richardson T, Hanks GE. Characteristics and quality assurance of a dedicated open 0.23 T MRI for radiation therapy simulation. Med Phys. 2002;29(11):2541-7.

5. Khoo VS, Dearnaley DP, Finnigan DJ, Padhani A, Tanner SF, Leach MO. Magnetic resonance imaging (MRI): considerations and applications in radiotherapy treatment planning. Radiotherapy and oncology : journal of the European Society for Therapeutic Radiology and Oncology. 1997;42(1):1-15. Epub 1997/01/01. PubMed PMID: 9132820.

6. Kapanen M, Collan J, Beule A, Seppala T, Saarilahti K, Tenhunen M. Commissioning of MRI-only based treatment planning procedure for external beam radiotherapy of prostate. Magnetic resonance in medicine : official journal of the Society of Magnetic Resonance in Medicine / Society of Magnetic Resonance in Medicine. 2013;70(1):127-35. Epub 2012/08/14. doi: 10.1002/mrm.24459. PubMed PMID: 22886780.

7. Alasti H, Islam M, Rink A, Carlone M, Breen S, Jaffray D. SU‐E‐J‐165: Shielding Design for An MR Guided Radiotherapy (MRgRT) Suite. Med Phys. 2013;40(6):189-.

8. Tadic T, Jaffray D, Winter J, Petropoulos L, Marle J, Sweitzer M, et al. WE‐G‐WAB‐07: Commissioning of a Novel Integrated Platform for Online MRIgRT. Med Phys. 2013;40(6):506-.

9. Nyholm T, Nyberg M, Karlsson MG, Karlsson M. Systematisation of spatial uncertainties for comparison between a MR and a CT-based radiotherapy workflow for prostate treatments. Radiat Oncol. 2009;4:54.

10. Devic S. MRI simulation for radiotherapy treatment planning. Med Phys. 2012;39:6701.

11. Kapanen M, Collan J, Beule A, Seppala T, Saarilahti K, Tenhunen M. Commissioning of MRI-only based treatment planning procedure for external beam radiotherapy of prostate. Magnetic resonance in medicine : official journal of the Society of Magnetic Resonance in Medicine / Society of Magnetic Resonance in Medicine. 2012. Epub 2012/08/14. doi: 10.1002/mrm.24459. PubMed PMID: 22886780.

12. Schubert K, Wenz F, Krempien R, Schramm O, Sroka-Perez G, Schraube P, et al. [Possibilities of an open magnetic resonance scanner integration in therapy simulation and three-dimensional radiotherapy planning]. Strahlentherapie und Onkologie : Organ der Deutschen Rontgengesellschaft [et al]. 1999;175(5):225-31. Epub 1999/06/05. PubMed PMID: 10356612.

13. Karlsson M, Karlsson MG, Nyholm T, Amies C, Zackrisson B. Dedicated magnetic resonance imaging in the radiotherapy clinic. International journal of radiation oncology, biology, physics. 2009;74(2):644-51. Epub 2009/05/12. doi: 10.1016/j.ijrobp.2009.01.065. PubMed PMID: 19427564.

14. Author. 2014.

15. Kanal E, Borgstede JP, Barkovich AJ, Bell C, Bradley WG, Felmlee JP, et al. American College of Radiology white paper on MR safety. American Journal of Roentgenology. 2002;178(6):1335-47 %@ 0361-803X.

16. Mutic S, Palta JR, Butker EK, Das IJ, Huq MS, Loo L-ND, et al. Quality assurance for computed-tomography simulators and the computed-tomography-simulation process: Report of the AAPM Radiation Therapy Committee Task Group No. 66. Med Phys. 2003;30:2762.

17. Comission J. Universal ProtocolDecember 5, 2013]. Available from: <http://www.jointcommission.org/standards_information/up.aspx>.

18. Ramaseshan R, Heydarian M. Comprehensive quality assurance for stereotactic radiosurgery treatments. Physics in medicine and biology. 2003;48(14):N199-205. Epub 2003/08/05. PubMed PMID: 12894984.

19. Kirby AM, Evans PM, Helyer SJ, Donovan EM, Convery HM, Yarnold JR. A randomised trial of supine versus prone breast radiotherapy (SuPr study): comparing set-up errors and respiratory motion. Radiotherapy and oncology : journal of the European Society for Therapeutic Radiology and Oncology. 2011;100(2):221-6. Epub 2010/12/17. doi: 10.1016/j.radonc.2010.11.005. PubMed PMID: 21159397.

20. Weinreb J, Bell R, Clarke G, Hedges L, Kneeland J, Price R. MRI Magnetic Resonance Imaging Quality Control Manual. American College of Radiology2004.

21. Jenkinson M. Fast, automated, N-dimensional phase-unwrapping algorithm. Magnetic Resonance in Medicine. 2003;49(1):193-7.

22. Och JG, Clarke GD, Sobol WT, Rosen CW, Mun SK. Acceptance testing of magnetic resonance imaging systems: report of AAPM Nuclear Magnetic Resonance Task Group No. 6. Med Phys. 1992;19:217.

23. Wang D, Doddrell DM, Cowin G. A novel phantom and method for comprehensive 3-dimensional measurement and correction of geometric distortion in magnetic resonance imaging. Magnetic Resonance Imaging. 2004;22(4):529-42. doi: <http://dx.doi.org/10.1016/j.mri.2004.01.008>.

24. Sumanaweera TS, Adler Jr JR, Napel S, Glover GH. Characterization of spatial distortion in magnetic resonance imaging and its implications for stereotactic surgery. Neurosurgery. 1994;35(4):696-704.

25. Baldwin LN, Wachowicz K, Fallone BG. A two-step scheme for distortion rectification of magnetic resonance images. Med Phys. 2009;36(9):3917-26. Epub 2009/10/09. PubMed PMID: 19810464.

26. Xu Y, Haacke EM. An iterative reconstruction technique for geometric distortion-corrected segmented echo-planar imaging. Magn Reson Imaging. 2008;26(10):1406-14. Epub 2008/09/12. doi: 10.1016/j.mri.2008.04.008. PubMed PMID: 18783907.

27. Huang H, Ceritoglu C, Li X, Qiu A, Miller MI, van Zijl PC, et al. Correction of B0 susceptibility induced distortion in diffusion-weighted images using large-deformation diffeomorphic metric mapping. Magn Reson Imaging. 2008;26(9):1294-302. Epub 2008/05/24. doi: 10.1016/j.mri.2008.03.005. PubMed PMID: 18499384; PubMed Central PMCID: PMC2612638.

28. Doran SJ, Charles-Edwards L, Reinsberg SA, Leach MO. A complete distortion correction for MR images: I. Gradient warp correction. Physics in medicine and biology. 2005;50(7):1343.

29. Tanner S, Finnigan D, Khoo V, Mayles P, Dearnaley D, Leach M. Radiotherapy planning of the pelvis using distortion corrected MR images: the removal of system distortions. Physics in medicine and biology. 2000;45(8):2117.

30. Author. 2013.

31. Author. 2012.

32. Determination of signal-to-noise ratio (SNR) in diagnostic magnetic resonance imaging. . NEMA standard publication: National Electrical Manufacturers Association.; 2008.

33. Determination of two dimensional geometric distortion in diagnostic magnetic resonance images. Washington, DC: National Manufacturers Electrical Association; 1990.

34. Price RR, Axel L, Morgan T, Newman R, Perman W, Schneiders N, et al. Quality assurance methods and phantoms for magnetic resonance imaging: Report of AAPM nuclear magnetic resonance Task Group No. 1. Med Phys. 1990;17(2):287-95.

35. Phantom Test Guidance for the ACR MRI Accreditation Program. American College of Radiology; 2005.

36. A Primer on Medical Device Interactions with Magnetic Resonance Imaging Systems: U.S. Food and Drug Administration; 1997. Available from: <http://www.fda.gov/MedicalDevices/DeviceRegulationandGuidance/GuidanceDocuments/ucm107721.htm>.

37. Catana C, van der Kouwe A, Benner T, Michel CJ, Hamm M, Fenchel M, et al. Toward Implementing an MRI-Based PET Attenuation-Correction Method for Neurologic Studies on the MR-PET Brain Prototype. Journal of Nuclear Medicine. 2010;51(9):1431-8. doi: 10.2967/jnumed.109.069112.

38. Keereman V, Fierens Y, Broux T, De Deene Y, Lonneux M, Vandenberghe S. MRI-based attenuation correction for PET/MRI using ultrashort echo time sequences. Journal of Nuclear Medicine. 2010;51(5):812-8.

39. Robson MD, Gatehouse PD, Bydder M, Bydder GM. Magnetic resonance: an introduction to ultrashort TE (UTE) imaging. Journal of computer assisted tomography. 2003;27(6):825-46.

40. Hsu SH, Cao Y, Huang K, Feng M, Balter JM. Investigation of a method for generating synthetic CT models from MRI scans of the head and neck for radiation therapy. Physics in medicine and biology. 2013;58(23):8419-35. Epub 2013/11/13. doi: 10.1088/0031-9155/58/23/8419. PubMed PMID: 24217183.

41. Johansson A, Karlsson M, Nyholm T. CT substitute derived from MRI sequences with ultrashort echo time. Med Phys. 2011;38(5):2708-14. doi: doi:<http://dx.doi.org/10.1118/1.3578928>.

42. Berker Y, Franke J, Salomon A, Palmowski M, Donker HCW, Temur Y, et al. MRI-Based Attenuation Correction for Hybrid PET/MRI Systems: A 4-Class Tissue Segmentation Technique Using a Combined Ultrashort-Echo-Time/Dixon MRI Sequence. Journal of Nuclear Medicine. 2012;53(5):796-804. doi: 10.2967/jnumed.111.092577.

43. Kim JH, Lee JS, Song I-C, Lee DS. Comparison of Segmentation-Based Attenuation Correction Methods for PET/MRI: Evaluation of Bone and Liver Standardized Uptake Value with Oncologic PET/CT Data. Journal of Nuclear Medicine. 2012;53(12):1878-82. doi: 10.2967/jnumed.112.104109.

44. Dixon WT. Simple proton spectroscopic imaging. Radiology. 1984;153(1):189-94.

45. Coombs BD, Szumowski J, Coshow W. Two-point Dixon technique for water-fat signal decomposition with B0 inhomogeneity correction. Magnetic Resonance in Medicine. 1997;38(6):884-9. doi: 10.1002/mrm.1910380606.

46. Hussain S, Perkins T. mDIXON is developed to simplify and accelerate liver MRI: White Paper 2011. Available from: <http://clinical.netforum.healthcare.philips.com/us_en/Explore/White-Papers/MRI/mDIXON-is-developed-to-simplify-and-accelerate-liver-MRI>.

47. Perkins T, Van Tilburg J, Herigault G, Eggers H, Duijndam A, Beck G, et al., editors. Preliminary clinical experience with a multiecho 2-point Dixon (mDixon) sequence at 3t as an efficient alternative for both the SAR-intensive acquired in-and out-of-phase chemical shift imaging as well as for 3D fat-suppressed T1-weighted sequence used for dynamic gadolinium-enhanced imaging. Proc Intl Soc Mag Reson Med; 2010.

48. Adamson J, Chang Z, Wang Z, Yin F-F, Cai J. Maximum intensity projection (MIP) imaging using slice-stacking MRIa). Med Phys. 2010;37(11):5914-20.

49. Jackson E, Bronskill M, Drost D, Och J, Pooley R, Sobol W, et al. Acceptance testing and quality assurance procedures for magnetic resonance imaging facilities. Report of MR Subcommittee Task Group 1. 2010. In: American Association of Medical Physics Online Report [Internet].

50. Wang D, Strugnell W, Cowin G, Doddrell DM, Slaughter R. Geometric distortion in clinical MRI systems: Part I: evaluation using a 3D phantom. Magnetic Resonance Imaging. 2004;22(9):1211-21. doi: <http://dx.doi.org/10.1016/j.mri.2004.08.012>.

51. Baldwin LN, Wachowicz K, Thomas SD, Rivest R, Fallone BG. Characterization, prediction, and correction of geometric distortion in 3 T MR images. Med Phys. 2007;34:388.

52. Redacted. 3.

53. Wang H, Balter J, Cao Y. Patient-induced susceptibility effect on geometric distortion of clinical brain MRI for radiation treatment planning on a 3T scanner. Physics in medicine and biology. 2013;58(3):465.

54. Seibert T, White N, Kim G, McDonald C, Farid N, Moiseenko V, et al. Distortion Inherent to Magnetic Resonance Imaging (MRI) Can Lead to Geometric Miss in Radiosurgery Planning. International Journal of Radiation Oncology• Biology• Physics. 2014;90(1):S97.

55. Cao J, Cui Y, Champ CE, Liu H, Xiao Y, Werner-Wasik M, et al. Determination of internal target volume using selective phases of a 4-dimensional computed tomography scan. Practical Radiation Oncology. 2012;2(3):186-92.

56. Hu Y, Caruthers SD, Low DA, Parikh PJ, Mutic S. Respiratory Amplitude Guided 4-Dimensional Magnetic Resonance Imaging. International Journal of Radiation Oncology*Biology*Physics. 2013;86(1):198-204.

57. Cai J, Chang Z, Wang Z, Paul Segars W, Yin F-F. Four-dimensional magnetic resonance imaging (4D-MRI) using image-based respiratory surrogate: A feasibility study. Med Phys. 2011;38(12):6384.

58. Remmert G, Biederer J, Lohberger F, Fabel M, Hartmann G. Four-dimensional magnetic resonance imaging for the determination of tumour movement and its evaluation using a dynamic porcine lung phantom. Physics in medicine and biology. 2007;52(18):N401.

59. Von Siebenthal M, Szekely G, Gamper U, Boesiger P, Lomax A, Cattin P. 4D MR imaging of respiratory organ motion and its variability. Physics in medicine and biology. 2007;52(6):1547.

60. Tryggestad E, Flammang A, Han-Oh S, Hales R, Herman J, McNutt T, et al. Respiration-based sorting of dynamic MRI to derive representative 4D-MRI for radiotherapy planning. Med Phys. 2013;40:051909.

61. Tsao J, Kozerke S. MRI temporal acceleration techniques. Journal of magnetic resonance imaging : JMRI. 2012;36(3):543-60. Epub 2012/08/21. doi: 10.1002/jmri.23640. PubMed PMID: 22903655.

62. Liney GP, Moerland MA, editors. Magnetic resonance imaging acquisition techniques for radiotherapy planning. Seminars in radiation oncology; 2014: Elsevier.

63. Lustig M, Donoho DL, Santos JM, Pauly JM. Compressed sensing MRI. Signal Processing Magazine, IEEE. 2008;25(2):72-82.

64. Redacted. 2.

**Appendix A: Daily Quality Assurance Worksheet**

1. **Safety II. Periodic Image Quality Test**

| \|  \| **Daily Result** \|  \| **Daily Result** \| \| --- \| --- \| --- \| --- \| \| Patient Alarm (Y/N) \|  \| Central Frequency (Hz) \|  \| \| RF Coils –  No Visual Defects (Y/N) \|  \| Transmitter Gain (dB) \|  \| \| Intercom System (Y/N) \|  \| Spatial Resolution \|  \| \| Compressor (Y/N) \|  \| Artifact (Y/N) \|  \| \| Helium Upper (%) \|  \| Type \|  \| \| Helium Lower (%) \|  \| PIQT Report  (no highlight) \|  \| \| Therapist Initials \|  \| |
| --- | --- | --- | --- | --- | --- | --- | --- | --- | --- | --- | --- | --- | --- | --- | --- | --- | --- | --- | --- | --- | --- | --- | --- | --- | --- | --- | --- | --- | --- | --- |

**III. ELPS Accuracy (+/- 2 mm specification)**

|  | **Offset (mm)** |
| --- | --- |
| Axial |  |
| Sagittal |  |
| Coronal |  |

**IV. 2D Geometric Distortion**

Record 2 mm bounding box dimensions in each plane and box dimension.

| **Orientation** |  | **Dimension (mm)** |
| --- | --- | --- |
| Transverse | Left-Right |  |
|  | Ant-post |  |
| Sagittal | Foot-head |  |
|  | Ant-post |  |
| Coronal | Foot-head |  |
|  | Left-Right |  |


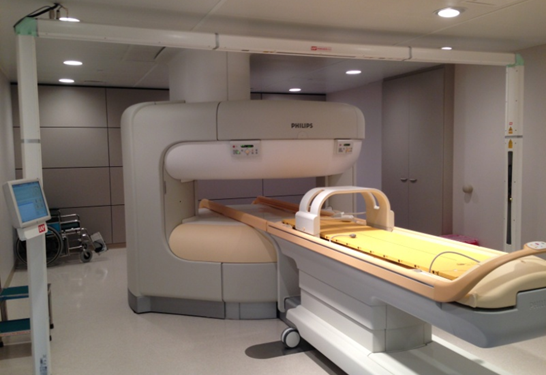


**b**

**c**

**e**

**a**

**g**

**d**

**f**

Fig. 1. (Left) Schematic of our MR-SIM suite and corresponding four-zone regions and their uses as recommended by the American College of Radiology. (Right) 1.0 T Philips Panorama High Field Open (HFO) Magnetic Resonance System radiation oncology dedicated MR-SIM and its components (a) external laser positioning system in-room console, (b) external laser positioning system (bridge), (c) vertical field magnet, (d) scanner’s integrated laser (i.e. light visor), (e) large body coil with solenoid technology, (f) MR-compatible indexed couchtop, and (g) patient support system trolley.


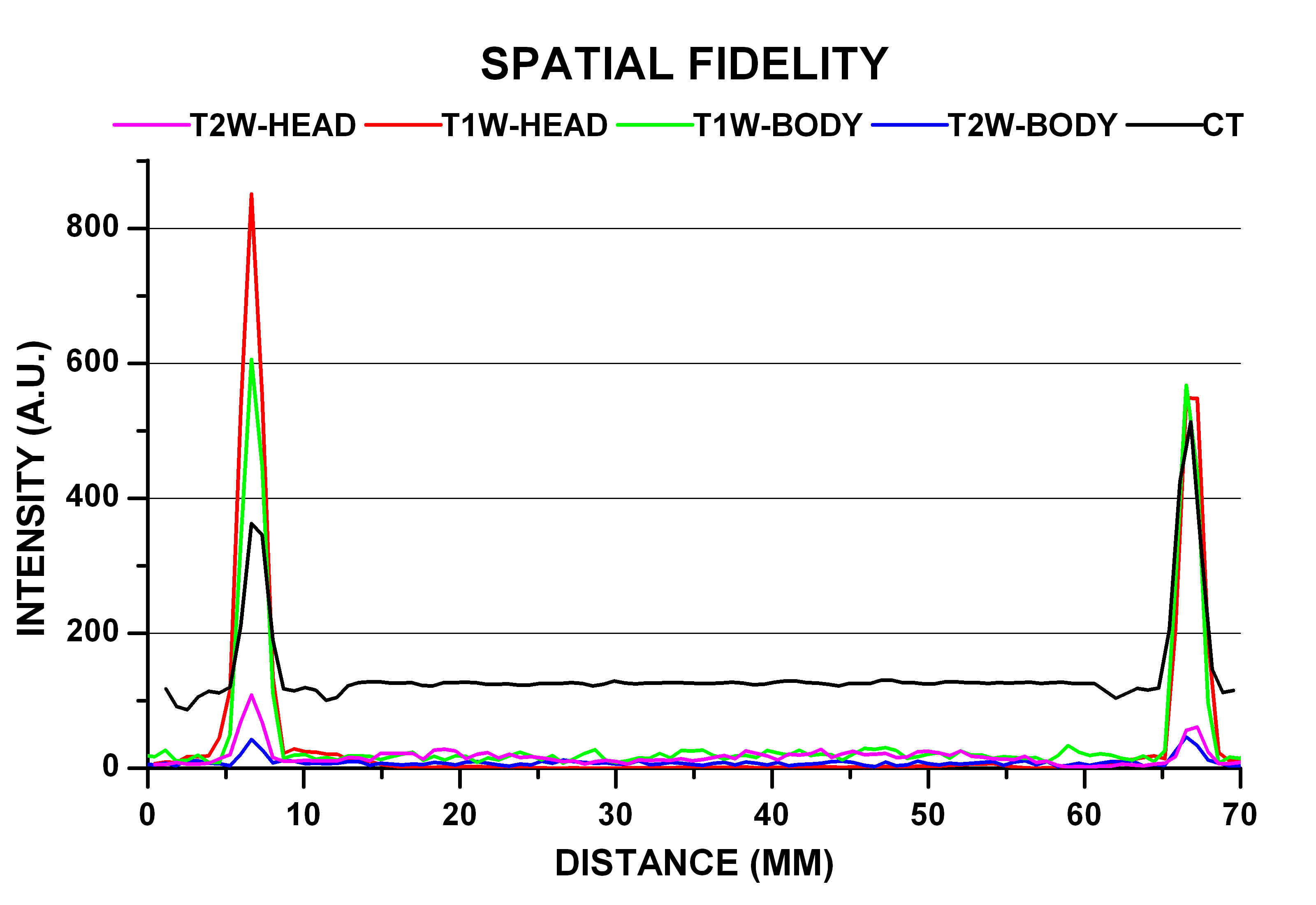


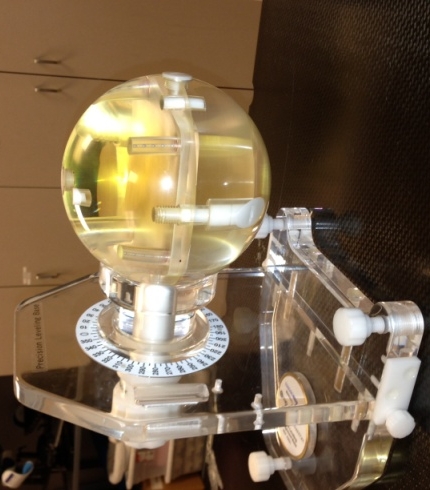

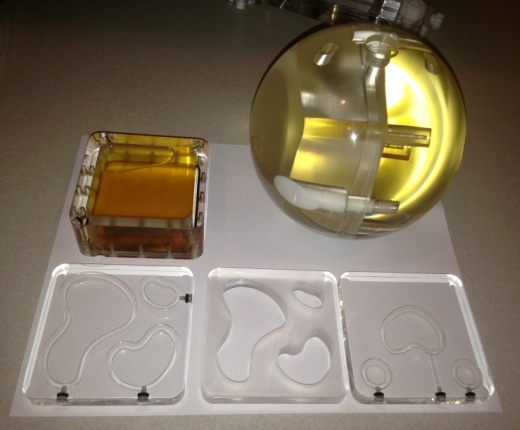


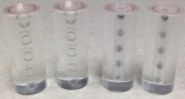


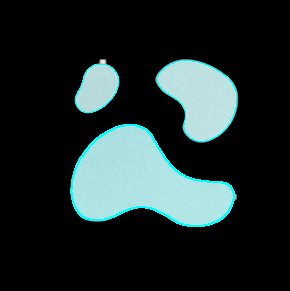

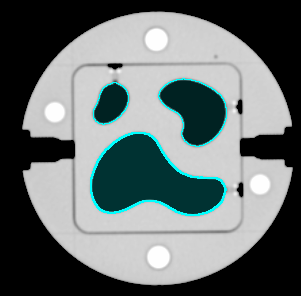

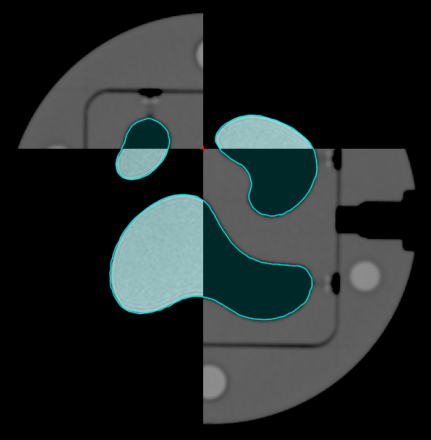

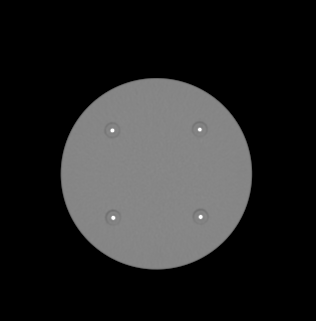

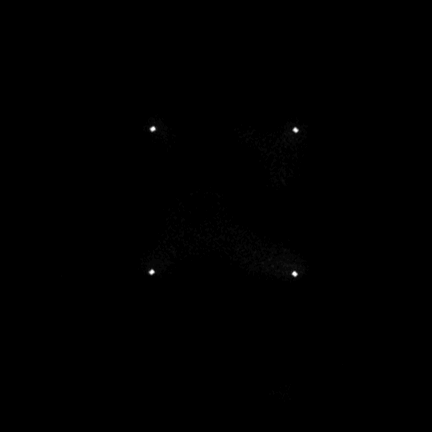


**d**

**L**

**R**

**ANT**

**POST**

**c**

**b**

**a**

**g**

**f**

**e**

**h**

Fig. 2. Lucy 3D QA Phantom used for volumetric, spatial, and registration comparisons between CT-SIM and MR-SIM. (a) Clockwise from the top left: phantom signal generator, Lucy phantom, MR and CT phantom inserts, (b) setup of the Lucy phantom using the manufacturer-provided leveling base. Red boxes indicate placement of CT marker cylinders used for spatial fidelity testing. Inset: MRI marker cylinders consisting of 2 mm oil beads (two left) and CT marker cylinders with 2 mm aluminum beads used for spatial testing (two right), (c) line profile analysis of the T1-weighted (T1W) and T2-weighted (T2W) datasets acquired using the head and body coils and the CT-SIM where the peaks indicate marker locations, (d) CT-SIM of the CT marker cylinders, (e) axial T1-weighted MR-SIM scan of the marker cylinders, (f) axial CT-SIM scan of the volumetric inserts, (g) axial T1-weighted MR-SIM scan of the volumetric inserts, and (h) image fusion of the CT-SIM andT1-weighted MR-SIM datasets. Legend: ANT = anterior, POST = posterior, L = left, R = right.


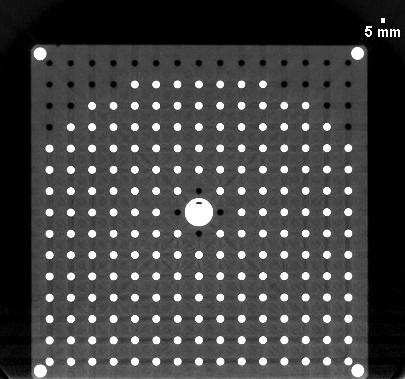

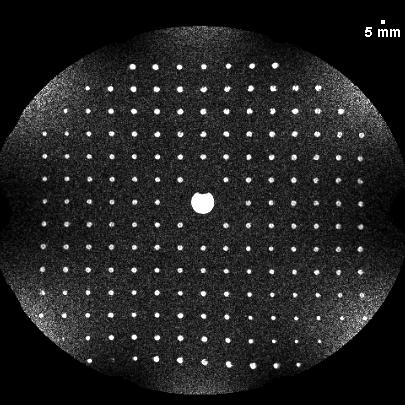


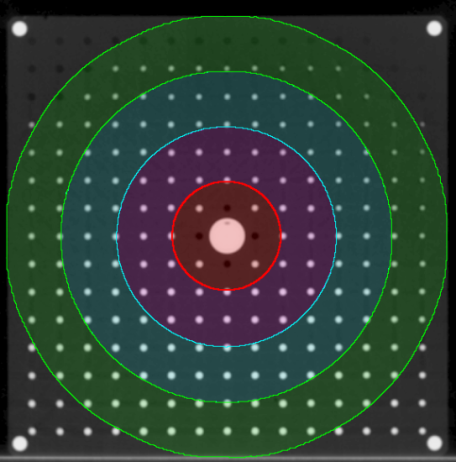


**C**

**RADIUS (CM): 0-5, 5-10, 10-15, 15-20**

**B**

**A**


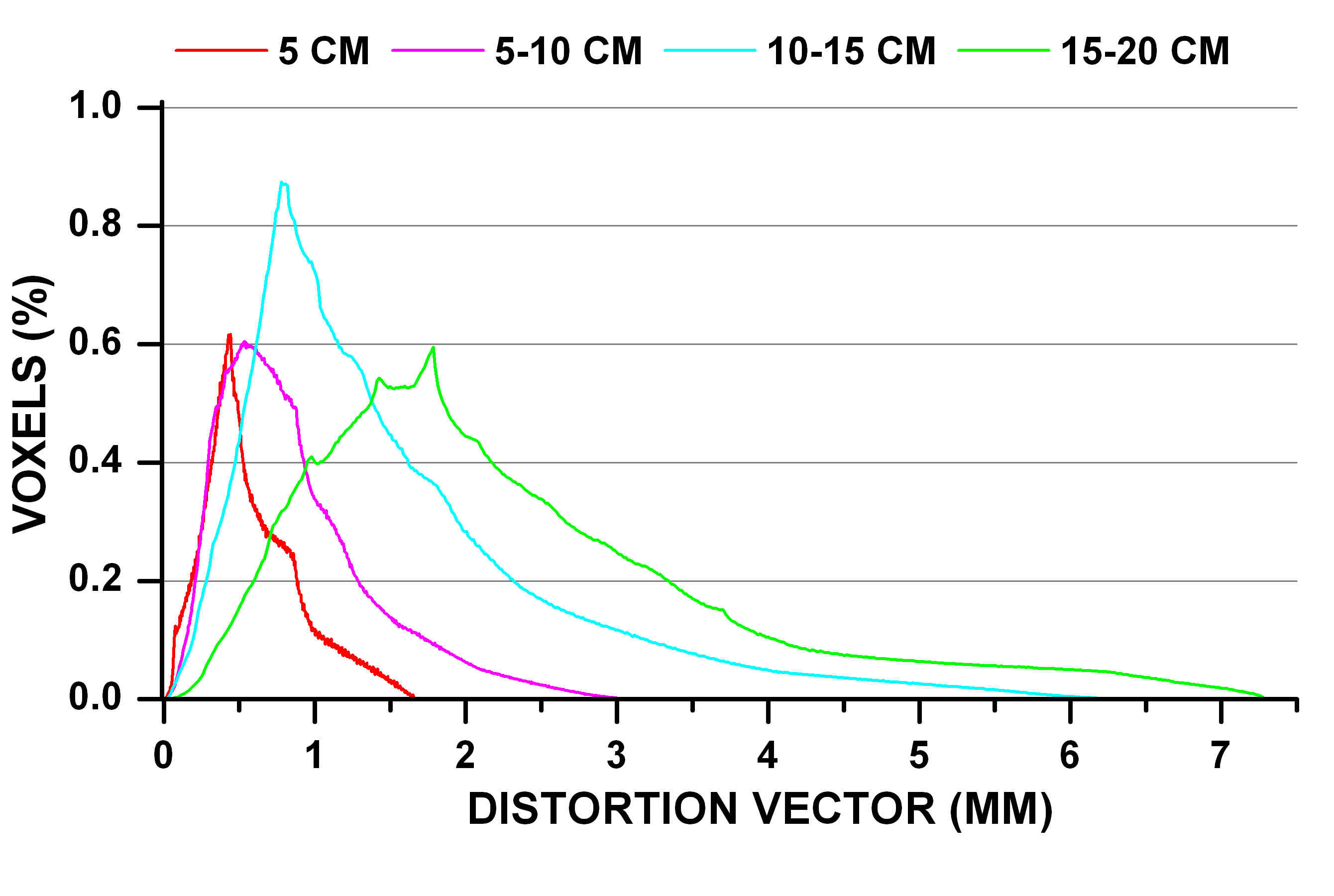


**D**

Fig. 3. (a) axial CT scan of the phantom demonstrating the capsule array, (b) 1.0T axial scan at similar slice location, (c) region of interests from isocenter used for distortion analysis between MR-SIM and CT-SIM, (d) distortion vector analysis between MR-SIM and CT-SIM at the different radii.


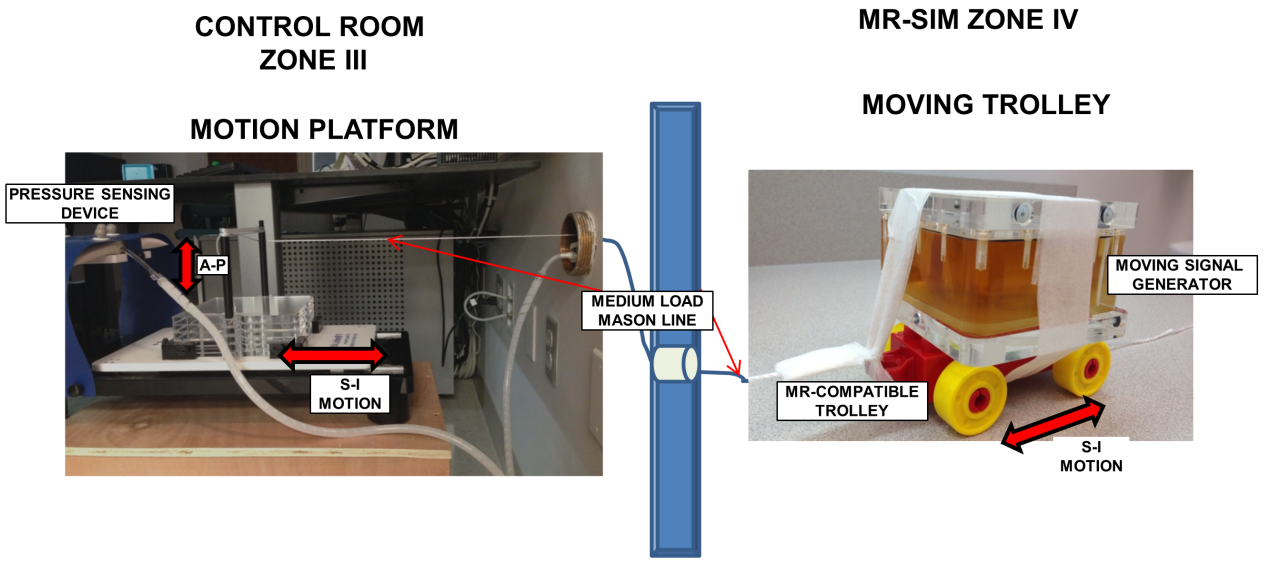


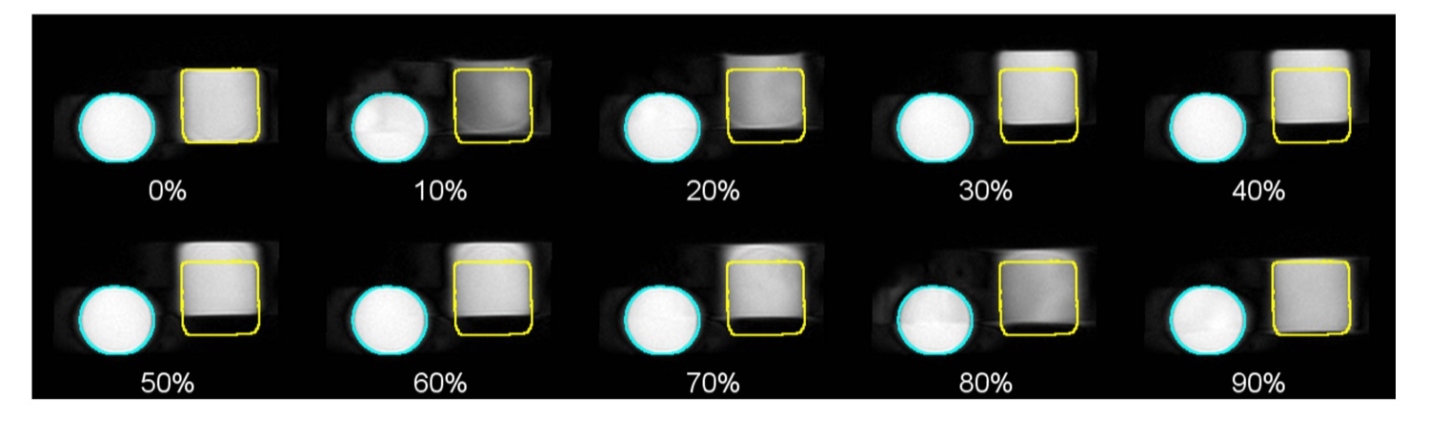


Fig. 4. (Top) Experimental setup for 4D-MRI phantom study, including the motion platform and components located in the control room that translate the MR-compatible trolley located in the MR-SIM bore. (Bottom) Coronal view for a ten-phase amplitude-based triggered 4D-MRI for a signal generator phantom translating ~2 cm in the superior-inferior direction (square object, sinusoidal breathing pattern, 3 second period). The percentages describe the breathing phases, with 0% representing end-inhale and 50% representing end-exhale. The round static signal generator is also shown in the field of view.


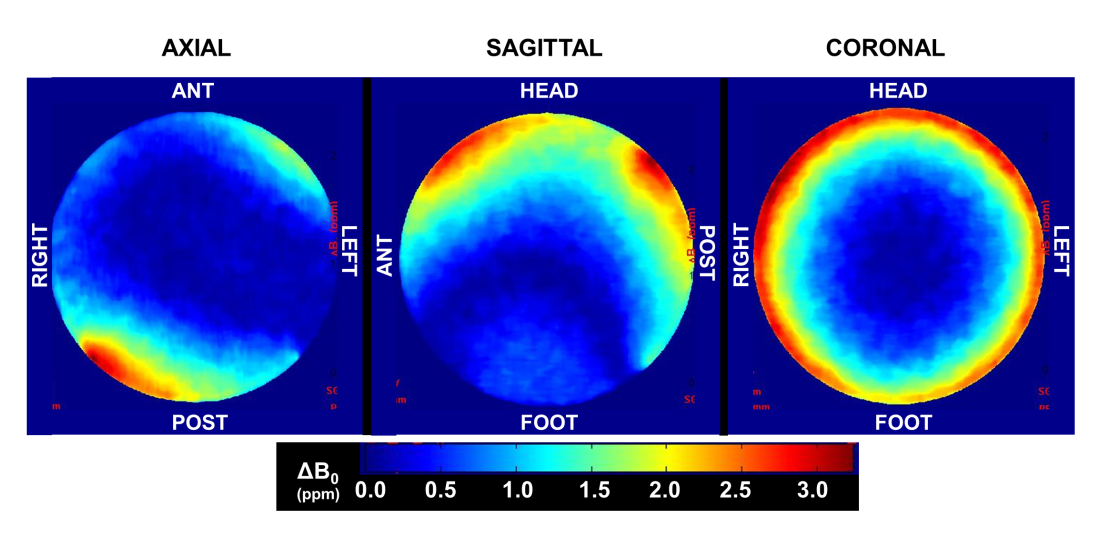


Fig. 5. Change in magnetic field inhomogeneity map characterized at time of acceptance. The volume root mean square was calculated to be 0.9 ppm over a 31 cm diameter spherical phantom volume, with an image reconstruction diameter of 35 cm.


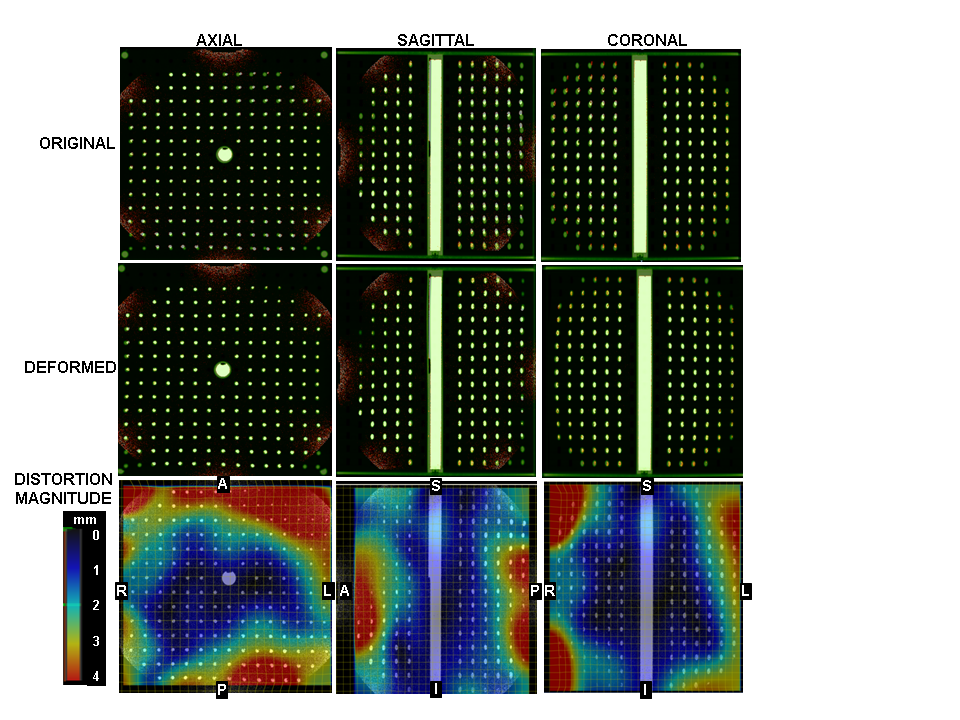


Fig. 6. Top row: Blended overlay of the original CT-SIM (green) and MR-SIM data (orange) prior to deformable image registration. Mismatches in marker location can be observed between datasets, particularly near the edges of the field of view. Middle row: Overlay of CT-SIM and MR-SIM data after deformable image registration, showing the resolution of image distortions. Bottom row: Vector distortion magnitude between the CT-SIM and MR-SIM datasets demonstrate the distortions occurring near the edges of the field of view. For reference, centroid-to-centroid marker spacing is 2.5 cm in-plane, 2.7 cm along the z-axis and the registration field of view was ~40 × 34 × 40 cm^3^ in the right-left, anterior-posterior, and superior-inferior, respectively.


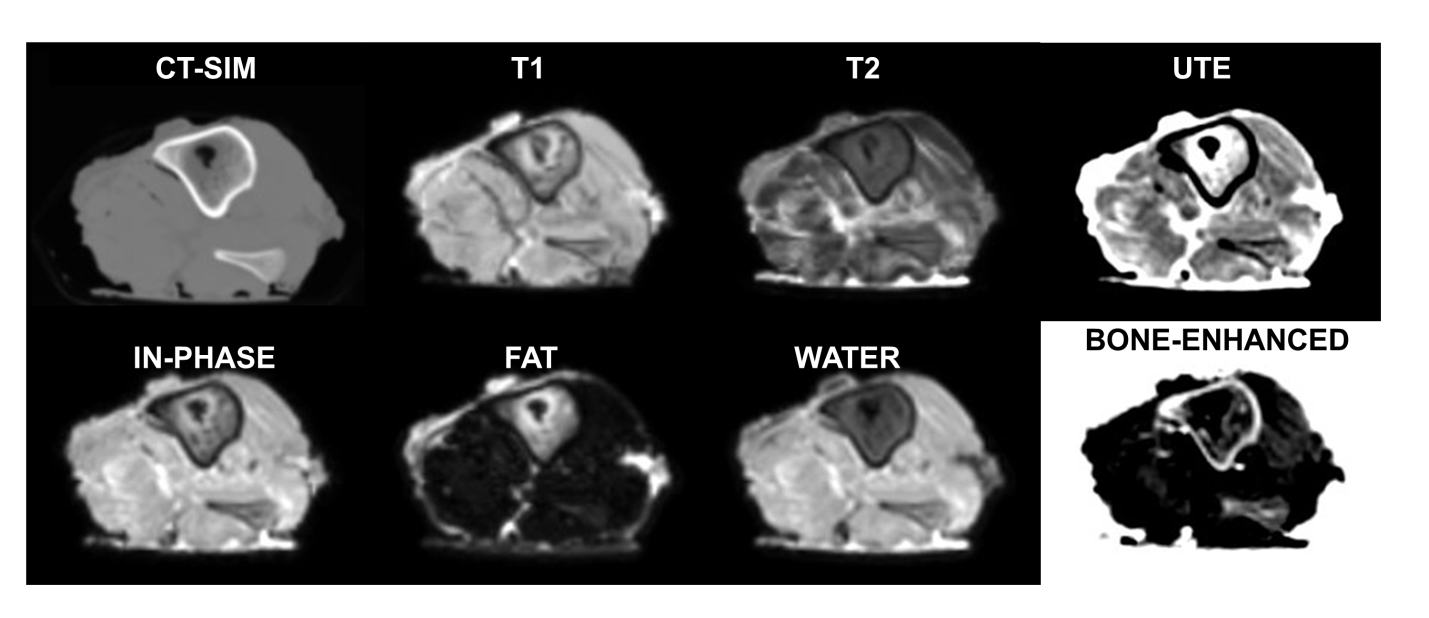


Fig. 7. Axial CT-SIM and 1.0T MR-SIM images for a porcine shank, including ultrashort echo time (UTE), water and fat images generated via DIXON acquistions, and a bone-enhanced image derived from the in-phase and UTE datasets.


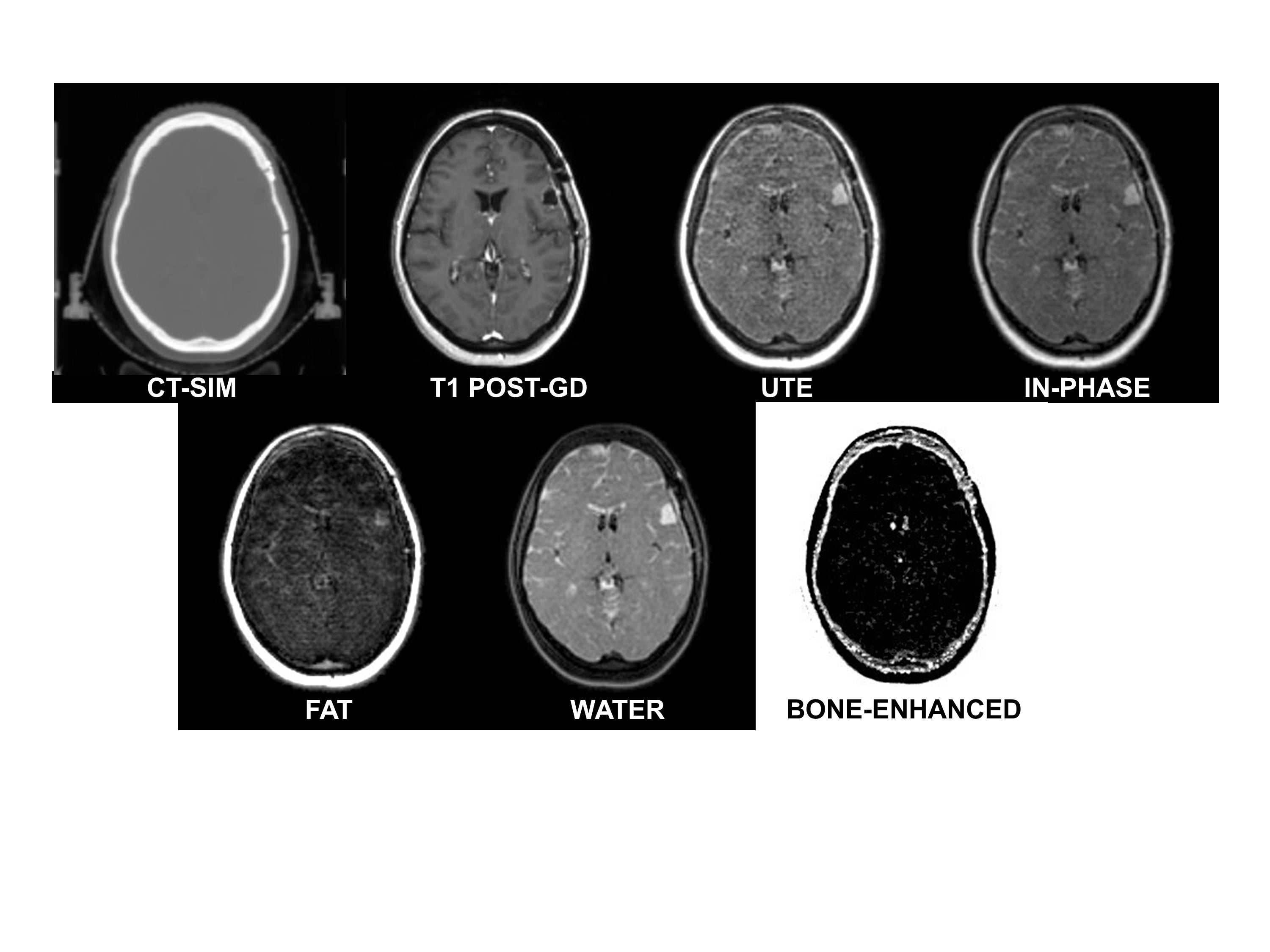


Fig. 8. Axial CT-SIM and 1.0T MR-SIM images for a post-surgical stereotactic radiosurgery patient with a left frontal metastasis, including a T1 post-Gd, ultrashort echo time (UTE), water and fat images generated via DIXON acquisitions, and a bone-enhanced image derived from the in-phase and UTE datasets.


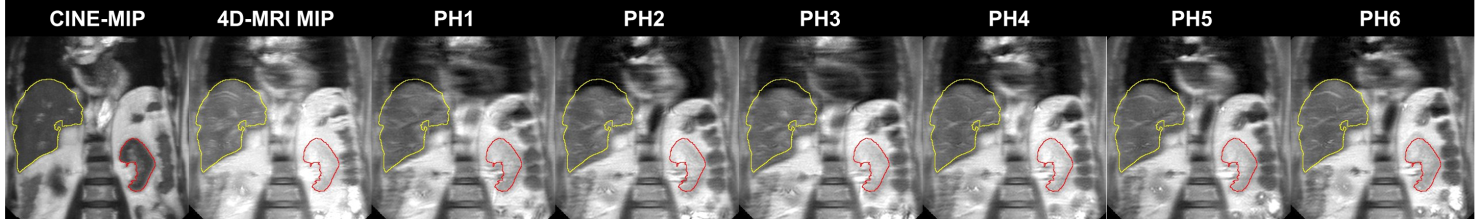


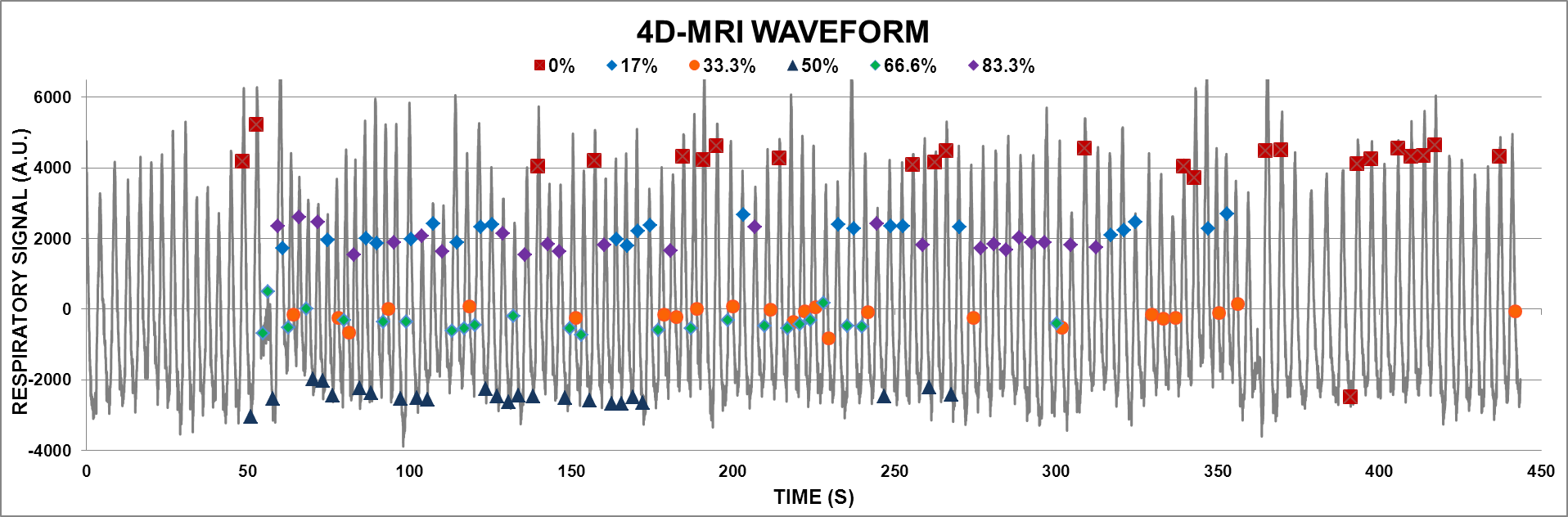


Fig. 9. (Top): Coronal view for 6-phase 4D-MRI for a liver cancer patient acquired in ~7.4 min. Contours derived from CINE-MIP. (Bottom) 4D-MRI waveform with respiratory tagging shown.

| **Patient Case** | **Imaging Coil** | **Immobilization Devices** | **Typical Sequences Acquired** |
| --- | --- | --- | --- |
| Pelvis   - Prostate - Gynecological | - Body coil: Medium-Extra Large - Integrated quadrature coil | - Square sponge under head - Angled sponge under knees/legs - Banded feet - Hands holding ring | **Whole pelvis**   - 3D T1-weighted Fast Field Echo - 3D T2-weighted Turbo Spin Echo   **Cone-down (higher resolution)**   - T2-weighted Turbo Spin Echo |
| Brain | Head coil | - Blue CIVCO Type-S Overlay board - CIVCO 5-point mask - Headrest “B” (clear /Silverman) - Angled sponge under knees/legs - Banded feet - Hands holding ring | - 3D T1-weighted - 3D T2-weighted Turbo Spin Echo - Axial T2-weighted Axial FLAIR |
| Cervical Spine | - Head coil - Body coil: Medium-Extra Large - Integrated quadrature coil | - Blue BodyFix bag (not pump) - Half circle leg sponge - Arms at sides - Feet neutral | - 3D T1-weighted - 3D T2-weighted Turbo Spin Echo - VISTA CLEAR - Balanced Fast Field Echo for head and neck region |
| Lumbar Spine | - Body coil: Medium-Extra Large - Integrated quadrature coil | - Blue BodyFix bag (not pump) - Half circle leg sponge - Arms at sides - Feet neutral | - 2D T1-weighted - 2D T2-weighted Turbo Spin Echo |

Table 1: Site-specific coils, immobilization, and imaging sequences optimized for MR-SIM implementation.
